# Conceptual framework for living with and beyond cancer: A systematic review and narrative synthesis

**DOI:** 10.1002/pon.5046

**Published:** 2019-03-25

**Authors:** Clair Le Boutillier, Stephanie Archer, Claire Barry, Alex King, Louise Mansfield, Catherine Urch

**Affiliations:** ^1^ Department of Surgery, Cardiovascular and Cancer Imperial College Healthcare NHS Trust London UK; ^2^ Department of Surgery & Cancer, Faculty of Medicine Imperial College London London UK; ^3^ Public Health and Primary Care University of Cambridge, Strangeways Research Laboratory Cambridge UK; ^4^ Department of Life Sciences Brunel University London London UK

**Keywords:** cancer, conceptual framework, living with and beyond, oncology, patient experience, systematic review

## Abstract

**Objective:**

The concept of living with and beyond cancer is now emerging in policy and literature. Rather than viewing this notion simply as a linear timeline, developing an agreed understanding of the lived experience of people affected by cancer will aid the development of person‐centred models of care.

**Methods:**

A systematic review was conducted. The review question was “What does the term ‘living with and beyond cancer’ mean to people affected by cancer?” The protocol for the review was preregistered in the PROSPERO database (PROSPERO CRD42017059860). All included studies were qualitative, so narrative synthesis was used to integrate descriptions and definitions of living with and beyond cancer into an empirically based conceptual framework.

**Results:**

Out of 2345 papers that were identified and 180 that were reviewed, a total of 73 papers were included. The synthesis yielded three interlinked themes: Adversity (realising cancer), Restoration (readjusting life with cancer), and Compatibility (reconciling cancer), resulting in the ARC framework.

**Conclusions:**

Three themes describe the experience of living with and beyond cancer: adversity, restoration, and compatibility. The ARC framework provides an empirically informed grounding for future research and practice in supportive cancer care for this population.

## 1 BACKGROUND

Around 2.5 million people are living with a cancer diagnosis in the United Kingdom and more than half of those people receiving cancer treatment will now live 10 years or more.^1^ While the concept of living with and beyond cancer is evident in international policy and literature, supporting people to live with and beyond cancer is complex because there are inconsistencies in understanding.^2^ For example, living with and beyond cancer is commonly used to refer to the process of coming to terms with, and overcoming, challenges associated with the effects of primary cancer treatment.^3^ On the other hand, people affected by cancer can lean towards a different meaning, whereby living with and beyond cancer begins at diagnosis.^4^ The need for clarity and an agreed understanding has therefore been identified to better understand the experiences and needs of those who are undergoing or have completed primary cancer treatment. This understanding can be used to develop person‐centred models of care and improve the long‐term quality of life for people who live with and beyond cancer.^5^

A conceptual framework addresses this need by providing a synthesised understanding of living with and beyond cancer and to date, no systematic review of evidence on conceptualising living with and beyond (all types of) cancer (LWBC) has been undertaken. The aim of this study was to identify and synthesise disparate accounts of LWBC into a thorough, empirically based conceptual framework, with a view to providing an empirical basis for future research and practice in cancer care.^6^

## 2 METHODS

The research aimed to draw together literature to explore the meaning of LWBC. The protocol for the review was preregistered in the PROSPERO database (PROSPERO CRD42017059860).

### 2.1 Eligibility criteria

The review sought to identify conceptual and empirical papers that explicitly developed a conceptualisation of living with and beyond any form of cancer. A conceptualisation of LWBC was defined as a theory, model, or framework, which emerged from an analysis of primary data on at least three participants.^7^ Debates on sample size and research quality continue; Pollio, Henley, and Thompson recommend, “although not a formal methodological rule, the situational diversity necessary for identifying thematic patterns is often provided by three to five interview transcripts.”8(p51) The aim of this study was to reflect the reality of participant accounts so a predefined understanding of LWBC was not used.

Studies were included if they

1. contained a conceptualisation of LWBC from which a succinct summary could be extracted;
2. presented an original model or framework (based on primary research) of LWBC;
3. presented primary research involving quantitative or qualitative data based on at least three participants;
4. were available in printed or downloadable form; and
5. were available in English.

Exclusion criteria were as follows:

1. studies focusing solely on living with the consequences of treatment, secondary symptoms, or dying;
2. studies focusing on health‐related quality of life that used a predefined definition; and
3. studies defining clinical remission criteria or recovery from cancer.

### 2.2 Search strategy and data sources

Three search strategies were used to identify relevant papers: electronic database searching, hand searching, and web‐based searching.

#### 2.2.1 Electronic database searching

Six bibliographic databases were searched: EMBASE; Health Management Information Consortium (HMIC); MEDLINE; PsycINFO; Scopus; CINAHL. All databases were searched from 2000 to March week 2 2017 using search terms identified in the title, abstract, and keywords. The search strategy was designed in OVID and modified for EBSCOhost and Elsevier and is shown in Table 1.

**Table 1:** Final search strategy

	Search Terms (Free Text Terms) Identified in the Title, Abstract, or Keywords	Concept
1	“cancer”	All cancer (not diagnosis specific)
2	(“life” OR “live” OR “lives” OR “living”) adj (“with” OR “beyond” OR “after”)	Living With and Beyond
3	“theor$” OR “framework” OR “model” OR “dimension” OR “paradigm” OR “concept$”	Understanding (truncated terms covering theory and conceptualisation)
4	1 AND 2 AND 3	
5	Limit to English Language AND Remove duplicates	

Initial scoping searches were completed using three databases (PsycINFO, Medline, and CINAHL) to narrow the key words and to test medical subject heading (MeSH) terms. Due to the specificity of the search (ie, patient experience and cancer as a disease), medical subject headings were not used, and search terms were refined and modified to optimise the balance between specificity and sensitivity. Limits were also placed on the protocol to ensure feasibility. For example, studies focusing on living with secondary symptoms or living with dying were not included.

#### 2.2.2 Hand searching

The tables of contents of journals which publish key articles (*British Journal of Cancer*, *Psycho‐Oncology*, and *Cancer Nursing)* were hand searched (from 2000 to May 2017). These journals were chosen because they were identified (eg, in database search) as having published research specific to LWBC. While secondary research was excluded from the review, existing systematic reviews^9^, ^10^, ^11^, ^12^, ^13^, ^14^, ^15^, ^16^ and literature reviews^17^ of living with and beyond cancer were also hand searched.

#### 2.2.3 Web‐based searching

An internet search using Google Scholar (scholar.google.co.uk) was conducted using the search term “living with and beyond cancer” to identify grey literature (ie, policy and practice guidance). The first 100 entries were reviewed. Specific cancer‐related and government websites (ie, National Cancer Research Institute) and the Macmillan Cancer Support internal evidence portal were also searched using the search term “living with and beyond cancer.” Articles citing included studies were searched using Web of Science (wok.mimas.ac.uk).

### 2.3 Data extraction

Duplicates were removed in Endnote, Version 7.^18^ Titles identified in the electronic search were screened, to identify those with possible relevance. Abstracts from relevant publications were reviewed, and where they appeared to meet the inclusion criteria, the full publication was obtained and assessed for eligibility. Of the 2280 abstracts identified in the database search, a random 15% (n = 342) were independently rated by authors C.L. and C.B. for eligibility.

One rater (C.L.) assessed the eligibility criteria for all 180 retrieved papers, with a random subsample of 85 papers independently rated for reliability by a second rater (C.B.). Acceptable concordance was predefined as agreement on at least 90% of ratings. A concordance of 92% was achieved. Reasons for exclusion were recorded, and disagreements were resolved through discussion or by a third rater (S.A.). For each included paper, the following data were extracted and tabulated: methodological approach, participant information and inclusion criteria, study location, and summary of main study findings.

### 2.4 Quality assessment

All included studies were qualitative, so quality was assessed using an established framework for assessing qualitative research evidence.^19^ The quality assessment covers the various stages and processes within qualitative enquiry, and the contribution, defensibility, rigour, and credibility of the study. One rater (C.L.) assessed the quality of all included studies, with a random 20% subsample of all papers (n = 15) independently rated by a second rater (C.B.). Consensus between raters was required, with differences in opinion on two of the 15 papers resolved through discussion. Spencer et al^19^ clarify that the quality framework is to be used as a heuristic guideline and so in order to make judgements about the overall quality of papers a point score was calculated using the quality framework.^19^ Each of the 18 items were weighted equally and rated “yes” (allocated 1 point) or “no” (allocated 0 points), giving a maximum quality rating of 18. The studies were divided into three groups; high quality was defined as a score of 13 or more, medium‐quality papers scoring 7 to 12, and low‐quality papers scoring 6 or less.

Quality assessment was not used to exclude papers given the debate on whether quality checklists rate the quality of the *study* or the quality of *reporting*.^20^ Instead, quality rating was used for sensitivity testing. For example, similarities and differences in results were explored across high‐quality studies as well as across all studies (high‐, medium‐, and low‐quality papers).

### 2.5 Data analysis

Narrative synthesis was used to synthesise the range and diversity of the key concepts of living with and beyond cancer identified in existing research. Narrative synthesis is an interpretive integration of qualitative findings that are themselves an interpretive synthesis of data. The narrative synthesis provides results that go beyond a description of the primary studies and provide a new interpretation and/or development of a new construct. This involves three stages: developing a preliminary synthesis, exploring relationships between studies and assessing the robustness of the synthesis.^21^

#### 2.5.1 Stage 1: developing a preliminary synthesis

To develop the preliminary synthesis, the main findings from each included study were analysed using inductive thematic analysis. This approach allows unexpected themes to emerge and does not restrict the investigation to predetermined concepts or prejudge the significance of concepts. One analyst (C.L.) extracted the data (themes and theme descriptions) from each included study into Microsoft Word tables. Analysis was then undertaken independently by two authors (C.L. and L.M.) who then discussed and compared their findings to develop a coding frame. Equal attention was paid to each data extract to identify initial codes, and codes were organised into one or several broader interpretive themes to fully capture their meaning.^22^ Thematic maps, that are a visual representation of the themes, were used to organise the themes by clustering all codes according to connections in the data and by considering the patterns and relationships between themes.^22^ Additional codes, refinements to the specifics of themes, and thematic patterns continued until theoretical saturation was achieved. Theoretical saturation occurred when the emergent themes had been fully explored and new data was easily accommodated within them.^22^ Themes needed to be present in at least two studies to be included in the synthesis, and the themes were confirmed as being representative of the literature by a second analyst (L.M.).

#### 2.5.2 Stage 2: exploring relationships between studies

Vote counting was conducted to identify similarities and differences between each study, including a subgroup analysis by country, cancer type, and stage of illness. Thematic vote counting was also conducted using codes developed in the thematic analysis for this cross case comparison.^21^

#### 2.5.3 Stage 3: assessing robustness of the synthesis

The preliminary conceptual framework was sent to an expert consultation panel to assess the robustness of the synthesis. The panel comprised 14 advisory committee members of the Living With and Beyond Cancer Programme (see www.imperial.nhs.uk/our‐services/cancer‐services/macmillan‐cancer‐partnership/living‐with‐and‐beyond‐cancer for further details) who had academic, clinical, or personal expertise about living with and beyond cancer. They were asked to comment on the general language and use of an acronym to describe the framework, the positioning of concepts within different hierarchical levels of the conceptual framework, to identify any important areas of LWBC which they felt had been omitted and to make any general observations. The preliminary conceptual framework was modified in response to these comments, to produce the final conceptual framework.

### 2.6 Ethics approval

The systematic review was conducted as part of a larger study, funded by Macmillan Cancer Support and hosted by Imperial College Healthcare NHS Trust. Ethical approval was obtained from the West Midlands–Black Country Research Ethics Committee and the Health Research Authority (REC reference 17/WM/0127).

## 3 RESULTS

**Figure 1:**
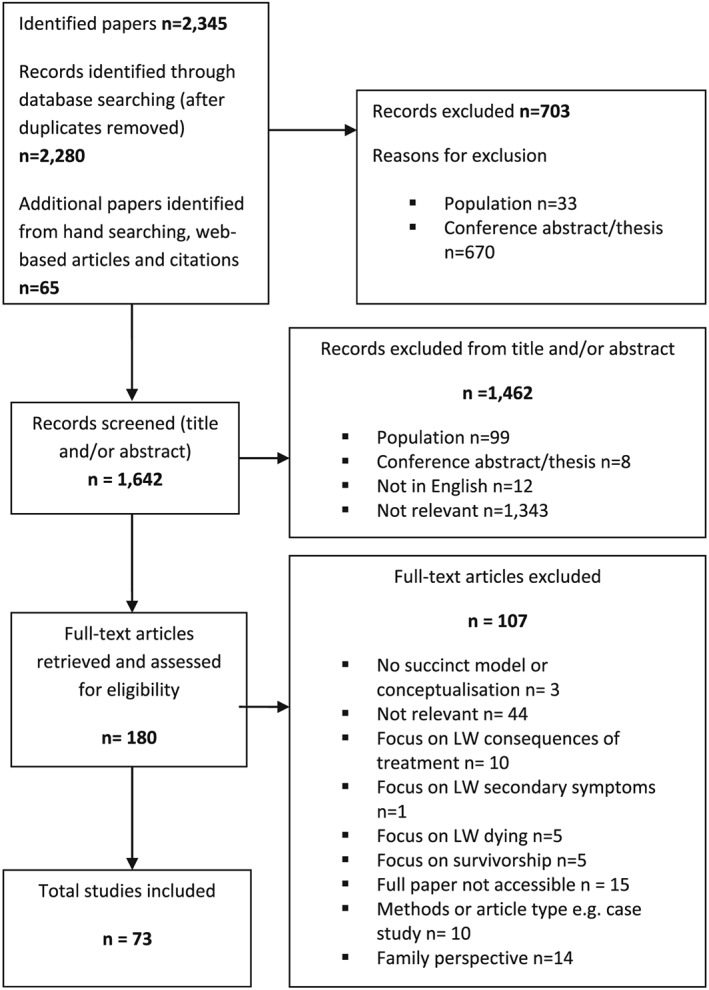
Flow chart to show assessment of eligibility of identified studies

A total of 73 studies focusing on the personal perspective of LWBC were identified for inclusion in the review. The flow diagram for the 73 included papers is shown in Figure 1 and Data S1 lists those papers that were included.

The 73 papers described studies conducted in 21 countries, across multiple cancer types. The sample sizes ranged from four to 156 participants with a mean sample size of 20. Data S2 lists the included study characteristics. The data extraction table provides full participant and setting details.

### 3.1 Quality assessment

The mean quality rating score for the 73 qualitative studies was 10.9 (range 3‐17). A quality rating score of 13 or above, indicating high quality, was obtained by 25 (34%) papers. A quality rating score of 7 to 12, indicating moderate quality, was obtained by 42 (58%) papers, and a quality rating score of 0 to 6, indicating low quality, was obtained by six (8%) papers. The quality ratings of each included study are reported in the data extraction table (Data S3).

### 3.2 Stage 1: developing a preliminary synthesis

Living with and beyond cancer is an experience that disrupts implicit assumptions about life and forces people to reconstruct their perspectives on self and future.^23^, ^24^, ^25^, ^26^, ^27^, ^28^, ^29^, ^30^, ^31^, ^32^, ^33^, ^34^, ^35^, ^36^, ^37^, ^38^ The disruption of living with and beyond cancer has been associated with both “relinquishing control”^39^ and “taking charge,”^40^ and while living with and beyond cancer is associated with loss and altered life,^41^, ^42^, ^43^, ^44^, ^45^, ^46^, ^47^, ^48^ included studies also report positive changed attitudes and views of life after a cancer diagnosis.^49^, ^50^, ^51^, ^52^, ^53^, ^54^, ^55^, ^56^, ^57^ The thematic analysis of the 73 included studies of conceptualisations of living with and beyond cancer yielded three interlinked common themes on how people make sense of their cancer experience: Adversity (realising cancer), Restoration (readjusting life with cancer) and Compatibility (reconciling cancer), resulting in the overarching ARC framework. The themes are interlinked because the experience of living with and beyond cancer is non‐linear. People affected by cancer can move backwards and forwards between themes as well as straddle across themes. Data S4 illustrates the ARC framework. Because of space limitations, a summary of each theme is provided and the subthemes are not elaborated in this article; the full coding framework is included in Data S5.

### 3.3 Theme 1: adversity—realising cancer

The theme “Adversity” refers to the distressing experience of reckoning with the life‐changing impact of a cancer diagnosis, symptoms and subsequent treatment. In particular, studies identified how the pathway to cancer diagnosis shaped the personal experience of living with and beyond cancer. For example, even before diagnosis, some participants expressed adversity with health care when they had to persist to get their symptoms noticed by professionals which delayed diagnosis, and for others, when professionals showed a lack of understanding and symptoms were misdiagnosed:

They kept telling me it was thrush and I kept telling them it wasn't; I said “I have had thrush before; it's not like this. ( Jefferies and Clifford58(p387) )

Communicating the diagnosis of cancer also had great impact on the experience. Adversity with healthcare was again experienced when participants were dissatisfied with the clinicians' communication style:

The Surgeon told me on his ward round, he said “you have got cancer and I'll be back later to explain,” I was on my own. At the end of the ward round I called the Consultant back to explain the diagnosis. ( France et al66(p345) )

The radiologist who saw the MRI said that the tumour is big, and there is great damage to the breast. You cannot imagine the way he told me that … this is not the way to talk. I left his office so distressed. All I thought about when I heard his talk is that the cancer is everywhere in my body, and there is no hope for me. ( Obeidat et al43(p308) )

Included studies also identify the overwhelming impact of a cancer diagnosis in terms of adversity experienced because of illness that included heightened awareness of the body, a challenge to identity and sense of self, and realisation of mortality. One participant describes how she will not be a part of the future as she had expected and hoped:

My oldest son got married last summer and I kept thinking I am never going to be a grandmother. I am surely going to die soon. I do not want to die. ( Sarenmalm et al59(p1121) )

Living with the effect of symptoms, that might include managing physical changes, emotional turmoil, loss of functional independence, reduced social well‐being, and financial distress, also causes adversity because of illness:

You could not go out and play, and nausea—it's not fun at all—and you'd like to take that away. But then there's the headaches, which you can live with, but some were so great that I just kind of said I do not want to live anymore. You kind of look at them and try and stage them, but they are all pretty bad cause if you look at tiredness, you are laying around and wasting your day, kind of not fun with those either. ( Woodgate and Degner60(p485) )

Physical, emotional, and social adversity is also experienced when managing the consequences of treatment. For example, dealing with changes in appearance, disrupted everyday roles, or social interactions. Other causes of social adversity include a shift in relationships, highlighted by concerns of burdening others, social isolation, changes in intimacy, the impact of providing support for others and, for some, the impact at work:

I do not always tell them (family) how bad things are, they know it cannot be cured, they know that, but you know, they'll come and say to me “how are you today?” “I am alright.” Even if I am not, because I do not like to worry them. ( Harley et al61(p348) )

Therefore, reliable support is highlighted as a significant need in the living with and beyond cancer experience:

I do not know what I expected or what sort of care I expected; not knowing what there is to be offered you know? Nobody's ever offered anything or said “oh well, we'll visit you” or anything like that … even if it was a little phone call to see how you are because you feel like you are … you have been told, you have been diagnosed, you are sent home and that's it. What do you do about it? That's how I felt. I was just thinking well what do I do about it? Angela. ( Reed and Corner62(p361) )

Included studies further highlight the adversity experienced because of healthcare. Some studies describe a “silent” health care system that provides limited time with healthcare professionals.^45^, ^63^ Others highlight the feeling of struggling and a loss of control due to the lack of participants' involvement in treatment planning:

Just people bossing you around and telling me what to do. I know it was for me own good, but people were just in your face all the time. ( Wicks and Mitchell42(p780) )

Loss of heath care and social support in the posttreatment phase is also reported to cause adversity. Oxlad et al found that following completion of primary treatment, participants described a void, as a result of a change in the level of healthcare support: “When it's all finished, it's empty isn't it? There's a void there.”54(p160) The ever‐present fear of recurrence will also contribute to ongoing stress:

I walk around with my nerves on edge, terrified of the slightest sign of pain, no matter where it might arise. ( Grimsbo et al64(p111) )

In your mind, whenever you are having your treatment, you are thinking, the treatment's keeping the cancer away. When the treatment stops, you are thinking, well what's keeping it away, now. ( Oxlad et al54(p162) )

### 3.4 Theme 2: restoration—readjusting life with cancer

The theme “Restoration” refers to the experience of readjusting or adapting one's life to manage the new context of cancer and recovery. Factors that were identified as part of the readjustment experience include confidence in health care, participation in treatment planning, and lifestyle changes:

I also have to play a part. I mean it's a partnership thing to manage this condition. A patient needs to be able to be educated to play a part, because not just leave it for the treatment here, play a part on your diet, play a part on what you do, play a part in everyday life to see what you can do to improve things. ( Beynon et al65(p173) )

Existing cancer knowledge and disease experience is another significant mediator for coping. For some, knowing that someone else has been through cancer treatment enables them to see a positive outcome:

You just have to cope, because no one is going to pick you up. You have to do it … I think possibly because it's just the knowledge sometimes of knowing that someone else has been through it and they are fine now. ( Fern et al26(pE34) )

Others draw on available sources of information and support to gain knowledge on what to expect from treatment:

A friend of the wife's [who] had a mastectomy eight months previous rang, came round to see me and told me what to expect when I went for the operation. ( France et al66(p346) )

Alongside access to information and accuracy of information, societal attitudes and stigma associated with cancer were found to be involved in the readjustment experience. For example, participants spoke about managing the challenges of “whispers in public” and about a desire to be able to appraise illness and talk about cancer openly:

People are not able to talk about it … it's this big monster you are carrying. ( Kelly and Dowling45(p40) )

Appraising illness and values in life were also identified as influencing the ability to readjust. Boehmke and Dickerson found that those who approached treatment as a transient experience to be handled in the short‐term were more able to move on with their lives. One participant described her illness as “something to get through, and I know in the end I will be fine.”24(p1124) The importance of social support and maintaining socially valued roles in restoration is also highlighted. Types of support identified by participants include relationships with family and friends and peer support and support groups:

The contact with my family and friends was very important and helped me not to lose faith. I needed to talk about it over and over again. ( Missel and Birkelund67[p299] )

There are other ladies in the same boat, sometimes we just sit and talk about it, it's about being in the same boat is not it? ( Davies and Sque68(p589) )

### 3.5 Theme 3: compatibility—reconciling cancer

The theme “Compatibility” refers to that aspect of the experience that relates to reconciling and rebalancing, creating new priorities and outlooks. For some participants, cancer is *just something else* in life, a contained, concrete medical event without complex ramifications:

I just took it as I would a toothache or whatever it was, except there was no pain involved. ( Foley et al39(p251) )

I just accept it as part of life. ( Foley et al39(p251) )

Cancer is cancer, what the hell? Like I say I am an old technician and so, if you got a broken piece, you take the broken piece out and replace it with a good piece. ( Pituskin et al49(p47) )

And for some, cancer was overshadowed by pre‐existing health conditions:

I certainly wasn't devastated by the fact that I'd got cancer … I think probably … one of the reasons was that perhaps the heart was taking precedence over it, in my mind. ( Appleton et al69(p76) )

Reconciling cancer means that problems assume other proportions whereby the focus turns to the more positive aspects of life and a shift in priorities, and that the present and day‐to‐day life are at the centre of things:

I think more about positive things like, really appreciate day‐to‐day life … and that you appreciate all the small things more, I think that is definitely true. ( Mattsson et al50(p1006) )

Trusson et al highlight a broader perspective of well‐being with the focus on what you have rather than what has been lost.^37^ Benefit finding is also found to support the process of reconciling and finding compatibility between life and cancer. Identified benefits include improved self‐esteem, better relations and a sense of connection, and a greater appreciation for life:

Going back, I would not change anything. I have often said that, and people look at you kind of funny. I had cancer but I would not change it because it's brought other things forward, it's brought the family closer. We have learned to deal with things a lot better. You have to experience what life gives you in order to be able to move on and be stronger with it. ( Pituskin et al49(p48) )

Kucukkaya found an increased self‐awareness, acceptance of old and renewed personality and increased appreciation of personal worth.^55^ Offering peer support and a willingness to help others is identified as another support mechanism in the search for compatibility where people affected by cancer guide others having had a shared experience:

We compare notes, we compare what medicine that we took, and what is the reaction that we had. So we empower them and make them understand that they are not alone in their fight. ( de Guzman et al70(p41) )

### 3.6 Stage 2: exploring the relationships between studies

All 73 studies were included in the vote‐counting process. For each dominant theme, papers were characterised using subthemes developed from the synthesis. Data S6 shows the vote counting for the subthemes of each of the three core themes. The “Adversity” subthemes present in the most studies were “life‐changing impact of diagnosis” (58 studies) and “impact of treatment” (48 studies). The “Restoration” subthemes most frequently identified were “importance of social support” (30 studies) and “lifestyle changes” (28 studies). The most frequent “Compatibility” subthemes were “benefit finding” (15 studies), “offering peer support and willingness to help others,” and “broader perspectives of well‐being” (both 11 studies each).

Overall, included studies of personal experiences of living with and beyond cancer made reference to Adversity, Restoration, and/or Compatibility. All three ARC themes were identified in 19 of the 73 studies (26%), with the strongest mapping for “Adversity” (94.5%) and the weakest mapping for “Compatibility” (34%). Contextual aspects such as country, study setting, participant (eg, age), or type of cancer did not produce any apparent differences within the ARC themes. High‐quality and low‐quality studies did not differ in their profiles. Eleven of the 25 studies assessed as high quality (scored 13+ out of a possible 18) identified all three themes in the findings.^26^, ^31^, ^37^, ^38^, ^53^, ^56^, ^71^, ^72^, ^73^, ^74^, ^75^ One study identified as the lowest quality (scored 6 out of a possible 18) also highlighted each of the ARC themes.^46^ Notably, four out of five studies that explored the lived experience of myeloma identified only with the concept of Adversity which reflects the nature of the condition's prognosis. Vote‐counting scores for each theme are included in Data S2.

### 3.7 Stage 3: assessing robustness of the synthesis

A response was received from six (43%) of the 14 consulted experts with academic, clinical, and/or personal expertise of living with and beyond cancer, who are advisory committee members of the Living With and Beyond Cancer Programme. Responses were themed under the following headings: conceptual (dangers of reductionism and limitations of stage models); structural (complete omissions and lack of emphasis or overemphasis on specific areas of LWBC); and language (too technical). In response to the experts' comments, the literature was revisited and the preliminary ARC framework was modified to have three interrelated rather than staged dominant categories. Some subcategories were repositioned within themes, and some category headings changed. Some responses identified areas of omission, such as the impact of how a cancer diagnosis is first communicated and the impact of cancer on employment. Alterations were made to include these as separate subcategories within the thematic analysis. Overall, the expert consultation process provided an additional validity check on the content and usefulness of the framework across different stakeholder groups in the health system.

## 4 CONCLUSIONS

Living with and beyond cancer is a concept that is used internationally in clinical practice and research. Despite this, there is little clarity with regard to what constitutes living with and beyond cancer from the perspective of patients and an agreed understanding of the concept is only just becoming established. The aim of the review and narrative synthesis was to obtain conceptual clarity about the personal experience of living with and beyond cancer. We identified three interlinked themes that describe the lived experience of cancer: Adversity, Restoration, and Compatibility, resulting in the ARC framework. The ARC framework provides an overarching synthesis of how people make sense of their cancer experience and is leading to a more nuanced understanding of what it means to live with and beyond cancer. To our knowledge, this is the first systematic review and narrative synthesis of personal perspectives, and the first empirical identification of an overarching conceptual framework for LWBC that can be used as a tool to define and operationalise the term.

The ARC framework complements and aligns constructively with existing literature on the experience of living with illness.^76^ For example, the ARC themes support the themes of biographical disruption identified by Bury: Adversity matches *Coping* that refers to methods used to manage the situation; Restoration matches *Strategy* that refers to the way in which people affected by chronic illness act to deal with it; and Compatibility matches *Style* that refers to the notion that different people have different attitudes towards illness.^77^ Charmaz, a medical sociologist and ethnographer, also presents the concept of “loss of self” as a central aspect of the experience of illness, beyond physical suffering. This refers to losing valued aspects of one's identity, due to a cascade of physical limitations and social changes.^78^ This account is mirrored in the “Adversity” and “Restoration” themes, which describe efforts to manage and contain the impact of diagnosis, symptoms, and treatment on personal identity, with the responses of one's social and health care context being a significant influence. Equally, Brennan proposes a clinically useful conceptual model of psychological adjustment in cancer, describing a continuous and iterative process of adapting one's mental models (of self and future etc) when cancer experience disconfirms one's previous implicit versions.^79^ The descriptions of a range of experiences within the “Adversity” theme align closely with this model. For example, even as people report reactions that are disparate on the surface (eg, cancer as “a massive shock” yet also cancer “not taking precedence”), they both derive from the same underlying process and thus can sit coherently within the same theme. In addition, the “Compatibility” theme relates to how people report a novel perspective on life and well‐being, with increased self‐awareness and changes in priorities, even if experiencing significant suffering. These experiences align well with the established concept of post‐traumatic growth, which Tedeschi and Calhoun describe in their review as “manifesting in a variety of ways, including an increased appreciation for life in general, more meaningful interpersonal relationships, an increased sense of personal strength, changed priorities, and a richer existential and spiritual life.”80(p1) Sherman et al also identify the diagnosis of cancer as a turning point in life, where cancer is recognised as a part of life, leading to the necessity of learning to live with cancer, and finally, to creating a new life after cancer.^72^ Furthermore, these qualitative and conceptual accounts are mirrored by quantitative studies of people LWBC that demonstrate increased psychosocial and interpersonal growth, despite poorer health and functioning.^81^ The ARC framework also extends the concept of post‐traumatic growth because the experience of living with and beyond cancer is individual and non‐linear, whereby people affected by cancer can move backwards and forwards between the ARC themes as well as straddle across themes.

### 4.1 Clinical and research implications

The ARC framework challenges more traditional cancer models because it is built from studies of personal experience. While a chronic disease model of care^82^ is widely adopted in the management of common chronic illness such as diabetes, depression, and heart failure (http://www.improvingchroniccare.org), the ARC framework questions the notion of chronicity associated with living with and beyond cancer and is a useful conceptual framework for translating the LWBC experience into shaping supportive cancer care. Like patient pathways that cross organisational boundaries, the ARC framework can also be used across all levels of the health system and in primary and secondary care.^83^ The ARC framework will be relevant in informing service design, patient advocacy and research, with a secondary role in direct clinical practice. The ARC framework describes the experience of living with and beyond cancer in patient‐centred terms, while at the same time aligning closely with established scientific concepts, models, and evidence. It also does not view personal experience as a predictable, linear process related solely to the clinical pathway. Taken together, these qualities can underpin the design of holistic supportive care services that are similarly patient‐centred, scientifically valid and non‐linear. For example, as “Adversity” (the work of realising the impact of cancer) is experienced and reexperienced and as the biopsychosocial impact of the illness unfolds, “patient education” needs to be delivered at multiple touch‐points rather than just clinical starting points. Also, the ARC framework can express significant aspects of cancer experience without resorting to unduly medicalising or reductive language. It can therefore empower patient representatives and advocates with a tool for promoting patient experience and a structure for its expression, thus increasing its impact. This may be especially relevant with regard to patient‐centred care, which is often a service priority but can be clouded by complexities regarding specific clinical protocols.^84^ Alongside, the ARC framework provides a foundation for structuring local guidelines and future policy, benchmarking clinical practice (eg, a basis for developing an accreditation process for services), and supporting staff development within existing practice competencies.^85^ In relation to research, the ARC framework can provide a core foundational structure as a starting point for investigation in novel and under‐researched areas, as well as challenging overly simple research questions. For instance, “Restoration” includes a significant social support dimension, and thus dyadic‐ or family‐wide rehabilitation may be more pertinent than more narrowly individual self‐management, though much more complex to research.^86^ Finally, at the level of clinical practice, the ARC framework can provide healthcare staff an evidence‐based tool for interpreting patients' narratives and guiding them appropriately. For instance, “Compatibility” highlights how cancer experience is often deeply transformative, and thus clinicians who hear a patient hoping that “after treatment everything will settle down and be the same again” can usefully reframe LWBC as “a new normal” that often involves both welcome and unwelcome changes. The ARC framework is informing clinical training in primary care Improving Access to Psychological Therapies services and the secondary care Clinical Nurse Specialist workforce. An example of ARC use in direct clinical practice is as a conversation guide for clinicians and patients completing the recovery package.^87^ For example, people are supported to identify interventions and assistance that would enable transition towards the readjustment and compatibility themes, as well as how they might be supported to manage any adversity, as part of their holistic needs assessment.

### 4.2 Study strengths and limitations

This is the first systematic review and narrative synthesis of personal perspectives on living with and beyond cancer. Until now, there has been lack of clarity around what it means to live with and beyond cancer. Adopting a transparent systematic review and narrative synthesis methodology addresses some of the criticisms regarding rigour and increases confidence in the findings. The robustness of the review was enhanced by three approaches to validating the framework, namely, the double rating of a proportion of papers to assess eligibility, double coding of included papers, and expert consultation on the preliminary framework. The strength of the conceptual framework can also be assessed on the strength of the evidence; with 67 of the 73 studies being rated as high or medium quality.

Secondly, by adopting broad and inclusive criteria on study context (eg, service setting and cancer pathway), while checking (using narrative synthesis methodology) that these do not unduly influence the results, this study allows the emergence of a broader account of patient experience. Thus, this framework is arguably more person‐centred and less subject to a priori influences and preconceptions of clinical parameters, policy priorities, or service realities. As a result, it can serve better as a solid basis for development in novel or underdeveloped areas (eg, a novel treatment or the experience of people with pre‐existing psychosocial vulnerabilities). It can also constructively challenge more established areas (eg, cancer rehabilitation) to reflect on whether the practices and solutions that have developed still align well with core, shared themes of personal experience.

Conversely, a potential limitation is that narrative synthesis is a secondary analysis of data based on existing interpretations by the authors of the original papers. As we necessarily accrue inferences and interpretations with every level of abstraction, there is potential for subtle or divergent accounts to be overlooked, reducing the richness of the account or inadvertently silencing some experiences. We safeguard against this through evaluating primary study quality, cross validating with clinicians and patients, and ensuring rigour throughout the research process. Nevertheless, we recognise that this account should be viewed as a heuristic framework, rather than as definitive and nomothetic. Similarly, it is important to note that while individual real‐world experiences can be accommodated by the ARC framework, the synthesis of existing studies was at group‐level and individual accounts go beyond the scope of this study.

A key challenge for health services is the lack of clarity around what constitutes living with and beyond cancer. The synthesis contributes to the understanding of living with and beyond cancer, and the emerging conceptual framework can be used to support clinical practice by identifying and responding to the needs of the personal experience of living with and beyond cancer.

## AUTHOR CONTRIBUTIONS

C.L. designed and coordinated the study, conducted data collection and analysis, and drafted the manuscript. S.A., C.B., and L.M. participated in the study design, data collection, and analysis. A.K. contributed to analysis. C.U. participated in the study design and data analysis.

All authors read and approved the final manuscript.

## CONFLICTS OF INTEREST

The authors declare they have no conflict of interest.

## Supporting information

Data S1: Included studies table (full reference list)
Data S2: Included study characteristics and vote counting of ARC themes
Data S3: Data extraction table
Data S4: The ARC Framework themes and sub‐themes
Data S5: Full coding framework
Data S6: Vote counting of ARC sub‐themes
