# Supplementary material for: Conceptual framework for living with and beyond cancer: A systematic review and narrative synthesis
Source: Psychooncology. 2019 Mar 25;28(5):948–59. doi: 10.1002/pon.5046 (PMC6594071; doi:10.1002/pon.5046)
Supplement: Supplementary file 1 — Data S1: Included studies table (full reference list) Data S2: Included study characteristics and vote counting of ARC themes Data S3: Data extraction table Data S4: The ARC Framework themes and sub‐themes Data S5: Full coding framework Data S6: Vote counting of ARC sub‐themes [file PON-28-948-s001.doc]

**SUPPORTING INFORMATION**

**Online data Supplement 1: Included studies table (full reference list)**

**Online data supplement 2: Included study characteristics and vote counting of ARC themes**

**Online data Supplement 3: Data extraction table**

**Online data Supplement 4: The ARC Framework themes and sub-themes**

**Online data Supplement 5: Full coding framework**

**Online data Supplement 6: Vote counting of ARC sub-themes**

**Online Data Supplement 1: Included Studies table (full reference list)**

| # | Study ID | Full reference |
| --- | --- | --- |
| 1 | PROUTY2006 | Prouty D, Ward-Smith P, Hutto CJ (2006) The lived experience of adult survivors of childhood cancer. Journal of Pediatric Oncology Nursing, 23 (3), 143-151 |
| 2 | WOODGATE 2003 | Woodgate RL, Degner LF (2003) Expectations and beliefs about children’s cancer symptoms: Perspectives of children with cancer and their families. Oncology Nursing Forum 30 (3), 479-491 |
| 3 | FERN2013 | Fern LA, Taylor RM, Whelan J, Pearce S, Grew T, Brooman K, Starkey C, Millington H, Ashton J, Gibson F (2013) The art of age-appropriate care. Reflecting on a conceptual model of the cancer experience for teenagers and young adults. Cancer Nursing 36 (5) E27-E38 |
| 4 | GRINYER2007 | Grinyer A (2007) The biographical impact of teenage and adolescent cancer. Chronic Illness 3, 265-277 |
| 5 | MATTSSON2007 | Mattsson E, Ringner A, Ljungman G, von Essen L (2007) Positive and negative consequences with regard to cancer during adolescence. Experiences two years after diagnosis. Psycho-Oncology 16, 1003-1009 |
| 6 | WICKS2010 | Wicks L, Mitchell A (2010) The adolescent cancer experience: loss of control and benefit finding. European Journal of Cancer Care 19, 778-785 |
| 7 | JONES2010 | Jones BL, Volker DL, Vinajeras Y, Butros L, Fitchpatrick C, Rossetto K (2010) The meaning of surviving cancer for Latino adolescents and emerging young adults. Cancer Nursing 33 (1), 74-81 |
| 8 | GRIMSBO2011 | Grimsbo GH, Finset A, Ruland CM (2011) Left hanging in the air. Experiences of living with cancer as expressed through email communications with oncology nurses. Cancer Nursing 34 (2), 107-116 |
| 9 | TRUSSON2016 | Trusson D, Pilnick A, Roy S (2016) A new normal?: Women’s experiences of biographical disruption and liminality following treatment for early stage breast cancer. Social Science and Medicine 151, 121-129 |
| 10 | McCANN2010 | McCann L, Illingworth N, Wengstrom Y, Hubbard G, Kearney N (2010) Transitional experiences of women with breast cancer within the first year following diagnosis. Journal of Clinical Nursing 19, 1969-1976 |
| 11 | LANDMARK2001 | Landmark BT, Strandmark M, Wahl AK (2001) Living with newly diagnosed breast cancer – the meaning of existential issues. A qualitative study of 10 women with newly diagnosed breast cancer, based on grounded theory. Cancer Nursing 24 (3), 220-226 |
| 12 | LANDMARK2002 | Landmark BT, Wahl A (2002) Living with newly diagnosed breast cancer: a qualitative study of 10 women with newly diagnosed breast cancer. Journal of Advanced Nursing 40 (1), 112-121 |
| 13 | OXLAD2008 | Oxlad M, Wade TD, Hallsworth L, Koczwara B (2008) ‘I’m living with a chronic illness, not… dying with cancer’: a qualitative study of Australian women’s self-identified concerns and needs following primary treatment for breast cancer. European Journal of Cancer Care 17, 157-166 |
| 14 | BOEHMKE2006 | Boehmke MM, Dickerson SS (2006) The diagnosis of breast cancer: Transition from health to illness. Oncology Nursing Forum 33 (6), 1121-1127 |
| 15 | OBEIDAT2012 | Fakhri Obedat R, Lally RM, Dickerson SS (2012) Arab American women’s lived experience with early-stage breast cancer diagnosis and surgical treatment. Cancer Nursing, 35 (4), 302-311 |
| 16 | SHERMAN2012 | Sherman DW, Rosedale M, Haber J (2012) Reclaiming life on one’s own terms: A grounded theory study of the process of breast cancer survivorship. Oncology Nursing Forum 39 (3), E258-E268 |
| 17 | KUCUKKAYA2010 | Kucukkaya PG (2010) An exploratory study of positive life changes in Turkish women diagnosed with breast cancer. European Journal of Oncology Nursing 14, 166-173 |
| 18 | CEBECI2012 | Cebeci F, Yangin HB, Tekeli A (2012) Life experiences of women with breast cancer in south western Turkey: A qualitative study. European Journal of Oncology Nursing 16, 406-412 |
| 19 | Da COSTA VARGENS2007 | Da Costa Vargens OM, Bertero CM (2007) Living with breast cancer. Its effect on the life situation and the close relationship of women in Brazil. Cancer Nursing 30 (6), 471-478 |
| 20 | GONZAGA2013 | Gonzaga MA (2013) Listening to the voices: an exploratory study of the experiences of women diagnosed and living with breast cancer in Uganda. Pan African Medical Journal 16:60. DOI: 10.11604/pamj.2013.16.60.2431 |
| 21 | LIAMPUTTONG2016 | Liamputtong P, Suwankhong D (2016) Living with breast cancer: the experience and meaning-making among women in southern Thailand. European Journal of cancer Care 25, 371-380 |
| 22 | DONOVAN2007 | Donovan T, Flynn M (2007) What makes a man a man? The lived experience of male breast cancer. Cancer Nursing 30 (6), 464-470 |
| 23 | NAYMARK2006 | Naymark P (2006) Male breast cancer: incompatible and incomparable. Journal of Men’s Health and Gender 3 (2), 160-165 |
| 24 | FRANCE 2000 | France L, Michie S, Barrett-Lee P, Brain K, Harper P, Gray J (2000) Male cancer: A qualitative study of male breast cancer. The Breast, 9, 343-348 |
| 25 | WILLIAMS2003 | Williams BG, Iredale R, Brain K, France E, Barrett-Lee P, Gray J (2003) Experiences of men with breast cancer: an exploratory focus group study. British Journal of Cancer 89, 1834-1836 |
| 26 | PITUSKIN2007 | Pituskin E, Williams B, Au H-J, Martin-McDonald K (2007) Experiences of men with breast cancer: a qualitative study. Journal of Men’s Health and Gender 4 (1), 44-51 |
| 27 | ROBERTS2009 | Roberts K, Clarke C (2009) Future disorientation following gynaecological cancer: women’s conceptualisation of risk after a life threatening illness. Health, Risk and Society 11 (4), 353-366 |
| 28 | LARANJEIRA2015 | Laranjeira CA, Leao PP, Leal I (2015) Temporal experience among women gynecological cancer survivors: A lifeworld perspective. Journal of women and social work 30 (2), 170-186 |
| 29 | THOMPSON2007 | Thompson K (2007) Liminality as a descriptor for the cancer experience. Illness, Crisis and Loss 15 (4), 333-351 |
| 30 | BOWES2002 | Bowes DE, Tamlyn D, Butler LJ (2002) Women living with ovarian cancer: Dealing with an early death. Health Care for Women International 23 (2), 135-148 |
| 31 | BURLES2013 | Burles M, Holtslander L (2013) “Cautiously optimistic that today will be another day with my disease under control”. Understanding women’s lived experiences of ovarian cancer. Cancer Nursing 36 (6), 436-444 |
| 32 | DING2015 | Ding Y, Hu Y, Hallberg IR (2015) Chinese women living with cervical cancer in the first 3 months after diagnosis. A qualitative study. Cancer Nursing 38 (1), 71-80 |
| 33 | JEFFERIES2009 | Jefferies H, Clifford C (2009) Searching. The lived experience of women with cancer of the vulva. Cancer Nursing 32 (6), E30-E36 |
| 34 | JEFFERIES 2012 | Jefferies H, Clifford C (2012) Invisibility. The lived experience of women with cancer of the vulva. Cancer Nursing 35 (5), 382-389 |
| 35 | GORDON2017 | Gordon H, LoBiondo-Wood G, Malecha A (2017) Penis Cancer. The lived experience. Cancer Nursing 40 (2), E30-E38 |
| 36 | APPLETON2013 | Appleton L, Goodlad S, Irvine F, Poole H, Wall C (2013) Patients’ experiences of living beyond colorectal cancer: A qualitative study. European Journal of Oncology Nursing, 17, 610-617 |
| 37 | SALAMONSEN2016 | Salamonsen A, Kiil MA, Kristoffersen AE, Stub T, Berntsen GR (2016) ‘My cancer is not my deepest concern’: life course disruption influencing patient pathways and health care needs among persons living with colorectal cancer. Patient Preference and Adherence 10, 1591-1600 |
| 38 | SHAHA2003 | Shaha M, Cox CL (2003) The omnipresence of cancer. European Journal of Oncology Nursing, 7 (3), 191-196 |
| 39 | MOLASSIOTIS2011 | Molassiotis A, Wilson B, Blair S, Howe T, Cavet J (2011) Living with multiple myeloma: experiences of patients and their informal caregivers. Supportive Cancer Care 19, 101-111 |
| 40 | POTRATA2011 | Potrata B, Cavet J, Blair S, Howe T, Molassiotis A (2011) Understanding distress and distressing experiences in patients living with multiple myeloma: an exploratory study. Psycho-oncology 20, 127-134 |
| 41 | KELLY2011 | Kelly M, Dowling M (2011) Patients’ lived experience of myeloma. Nursing Standard 25 (28), 38-44 |
| 42 | STEPHENS2014 | Stephens M, McKenzie H, Jordens CFC (2014) The work of living with a rare cancer: multiple myeloma. Journal of Advanced Nursing 70 (12), 2800-2809 |
| 43 | VLOSSAK2008 | Vlossak D, Fitch MI (2008) Multiple myeloma: The patient’s perspective, Canadian Oncology Nursing Journal, 141-146 DOI: 10.5737/1181912x183141145 |
| 44 | BEYNON2015 | Beynon T, Selman L, Radcliffe E, Whittaker S, Child F, Orlowska D, Morgans C, Morris S, Harding R (2015) ‘We had to change to single beds because I itch in the night’: a qualitative study of the experiences, attitudes and approaches to coping of patients with cutaneous T-cell lymphoma. British Journal of Dermatology 173, 83-92 |
| 45 | LEAL2015 | Leal I, Engebretson J, Cohen L, Rodriguez A, Wangyal T, Lopez G, Chaoul A (2015) Experiences of paradox: a qualitative analysis of living with cancer using a framework approach. Psycho-oncology 24, 138-146 |
| 46 | KOENIGSMANN2006 | Koenigsmann M, Koehler K, Regner A, Franke A, Frommer J (2006) Facing mortality: A qualitative in-depth interview study on illness perception, lay theories and coping strategies of adult patients with acute leukemia 1 week after diagnosis. Leukemia Research 30, 1127-1134 |
| 47 | ANDREASSEN2006 | Andreassen S, Randers I, Naslund E, Stockeld D, Mattiasson A-C (2006) Patients’ experiences of living with oesophageal cancer. Journal of Clinical Nursing 15, 685-695 |
| 48 | ROING2009 | Roing M, Hirsch J-M, Holmstrom I, Schuster M (2009) Making new meanings of being in the world after treatment for oral cancer. Qualitative Health Research, 19 (8), 1076-1086 |
| 49 | VAN DIJK2010 | Van Dijk J, Oostrom KJ, Huisman J, Moll AC, Cohen-Kettenis PT, Ringens PJ, Imhof SM (2010) Restrictions in daily life after retinoblastoma from the perspective of the survivors. Pediatric Blood Cancer 54, 110-115 |
| 50 | KENNESARENMALM2009 | Kenne sarenmalm E, Thoren-jonsson A-L, Gaston-Johansson F, Ohlen J (2009) Making sense of living under the shadow of death: Adjusting to a recurrent breast cancer illness. Qualitative Health Research 19 (*), 1116-1130 |
| 51 | KJORVENHAUG2014 | Kjorven Haug SH, Danbolt LJ, Kvigne K, DeMarinis V (2016) Older people with incurable cancer: Existential meaning-making from a life-span perspective. Palliative and Supportive Care 14, 20-32 |
| 52 | MISSEL2011 | Missel M, Birkelund R (2011) Living with incurable oesophageal cancer: A phenomenological hermeneutical interpretation of patient stories. European Journal of Oncology Nursing, 15, 296-301 |
| 53 | HAGGLUND2015 | Hagglund M, Bolin P, Koch S (2015) Living with lung cancer – Patients’ experiences as input to eHealth service design. MEDINFO 391-395. DOI: 10.3233/978-1-61499-564-7-391 |
| 54 | APPLETON2015 | Appleton L, Wyatt D, Perkins E, Parker C, Crane J, Jones A, Moorhead L, Brown V, Wall C, Pagett M (2015) The impact of prostate cancer on men’s everyday life. European Journal of Cancer Care 24, 71-84 |
| 55 | CAYLESS2009 | Cayless S, Forbat L, Illingworth N, Hubbard G, Kearney N (2010) Men with prostate cancer over the first year of illness: their experiences as biographical disruption. Supportive Care in Cancer 18, 11-19 |
| 56 | ERVIK2010 | Ervik B, Nordoy T, Asplund K (2010) Hit by waves – living with local advanced or localized prostate cancer treated with endocrine therapy or under active surveillance. Cancer Nursing 33 (5), 382-389 |
| 57 | JONSSON2010 | Jonsson A, Aus G, Bertero C (2010) Living with a prostate cancer diagnosis: a qualitative 2-year follow-up study. The Aging Male, 13 (1), 25-31 |
| 58 | CARTER2004 | Carter H, MacLeod R, Brander P, McPherson K (2004) Living with a terminal illness: patients’ priorities. Journal of Advanced Nursing 45 (6), 611-620 |
| 59 | DAVIES2002 | Davies M, Sque M (2002) Living on the outside looking in: a theory of living with advanced breast cancer. International Journal of Palliative Nursing 8 (12), 583-590 |
| 60 | HARLEY2015 | Harley C, Pini S, Bartlett YK, Velikova G (2015) Defining chronic cancer: patient experiences and self-management needs. BMJ Supportive and Palliative Care 5, 343-350 |
| 61 | HOULDIN2006 | Houldin AD, Lewis FM (2006) Salvaging their normal lives: A qualitative study of patients with recently diagnosed advanced colorectal cancer. Oncology Nursing Forum 33 (4), 719-725 |
| 62 | LA COUR2009 | La Cour K, Johannessen H, Josephsson S (2009) Activity and meaning-making in the very day lives of people with advanced cancer. Palliative and Supportive Care 7, 469-479 |
| 63 | LEVY2015 | Levy A, Cartwright T (2015) Men’s strategies for preserving emotional well-being in advanced prostate cancer: An interpretive phenomenological analysis. Psychology & Health, 30 (10), 1164-1182 |
| 64 | MAHER2011 | Maher K, De Vries K (2011) An exploration of the lived experiences of individuals with relapsed multiple myeloma. European Journal of Cancer Care, 20, 267-275 |
| 65 | NISSIM2012 | Nissim R, Rennie D, Fleming S, Hales S, Gagliese L, Rodin G (2012) Goals set in the land of the living/dying: A longitudinal study of patients living with advanced cancer. Death Studies 36 (4), 360-390 |
| 66 | REED2015 | Reed E, Corner J (2015) Defining the illness trajectory of metastatic breast cancer. BMJ Supportive and Palliative Care 5, 358-365 |
| 67 | REEVE2009 | Reeve J, Lloyd-Williams M, Payne S, Dowrick C (2009) Insights into the impact of clinical encounters gained from personal accounts of living with advanced cancer. Primary Health Care Research and Development 10, 357-367 |
| 68 | REEVE2010 | Reeve J, Lloyd-Williams M, Payne S, Dowrick C (2010) Revisiting biographical disruption: Exploring individual embodied illness experience in people with terminal cancer. Health 14 (2), 178-195 |
| 69 | SINDING2002 | Sinding C, Gray R, Fitch M, Greenberg M (2002) Staging breast cancer, rehearsing metastatic disease. Qualitative Health Research, 12 (1), 61-73 |
| 70 | WINTERLING2004 | Winterling J, Wasteson E, Glimelius B, Sjoden P-O, Nordin K (2004) Substantial changes in life. Perceptions in patients with newly diagnosed advanced cancer and their spouses. Cancer Nursing 27 (5), 381-388 |
| 71 | DE GUZMAN2013 | de Guzman AB, Jimenez CB, Jocson KP, Junio AR, Junio DE, Jurado JBN, Justiniano ABF (2013) This too shall pass: A grounded theory study of Filipino cancer survivorship. Journal of Holistic Nursing 31 (1), 35-46 |
| 72 | FOLEY2006 | Foley KL, Farmer DF, Petronis VM, Smith RG, Mcgraw S, Smith K, Carver CS, Avis N (2006) A qualitative exploration of the cancer experience among long-term survivors: Comparisons by cancer type, ethnicity, gender, and age. Psyho-oncology 15, 248-258 |
| 73 | HUBBARD2011 | Hubbard G, Forbat L (2012) Cancer as biographical disruption: constructions of living with cancer. Supportive Care in Cancer 20, 2033-2040 |

***Online data Supplement 2: Included study characteristics and vote counting of ARC themes***

| **#** | **Study ID** | **Country** | **Cancer type(s)** | **Adversity: Realising cancer** | **Restoration: Readjusting life with cancer** | **Compatibility: Reconciling cancer** |
| --- | --- | --- | --- | --- | --- | --- |
| **1**  **2**  **3**  **4**  **5**  **6**  **7**  **8**  **9**  **10**  **11**  **12**  **13**  **14**  **15**  **16**  **17**  **18**  **19**  **20**  **21**  **22**  **23**  **24**  **25**  **26**  **27**  **28**  **29**  **30**  **31**  **32**  **33**  **34**  **35**  **36**  **37**  **38**  **39**  **40**  **41**  **42**  **43**  **44**  **45**  **46**  **47**  **48**  **49**  **50**  **51**  **52**  **53**  **54**  **55**  **56**  **57**  **58**  **59**  **60**  **61**  **62**  **63**  **64**  **65**  **66**  **67**  **68**  **69**  **70**  **71**  **72**  **73** | **PROUTY2006**  **WOODGATE2003**  **FERN2013**  **GRINYER2007**  **MATTSSON2007**  **WICKS2010**  **JONES2010**  **GRIMSBO2011**  **TRUSSON2016**  **McCANN2010**  **LANDMARK2001**  **LANDMARK2002**  **OXLAD2008**  **BOEHMKE2006**  **OBEIDAT2012**  **SHERMAN2012**  **KUCUKKAYA2010**  **CEBECI2012**  **DaCOSTAVARGENS2007**  **GONZAGA2013**  **LIAMPUTTONG2016**  **DONOVAN2007**  **NAYMARK2006**  **FRANCE 2000**  **WILLIAMS2003**  **PITUSKIN2007**  **ROBERTS2009**  **LARANJEIRA2015**  **THOMPSON2007**  **BOWES2002**  **BURLES2013**  **DING2015**  **JEFFERIES2009**  **JEFFERIES 2012**  **GORDON2017**  **APPLETON2013**  **SALAMONSEN 2016**  **SHAHA2003**  **MOLASSIOTIS2011**  **POTRATA2011**  **KELLY2011**  **STEPHENS2014**  **VLOSSAK2008**  **BEYNON2015**  **LEAL2015**  **KOENIGSMANN2006**  **ANDREASSEN2006**  **ROING2009**  **VAN DIIJK2010**  **KENNESARENMALM2009**  **KJORVEN HAUG2014**  **MISSEL2011**  **HAGGLUND2015**  **APPLETON2015**  **CAYLESS2009**  **ERVIK2010**  **JONSSON2010**  **CARTER2004**  **DAVIES2002**  **HARLEY2015**  **HOULDIN2006**  **LA COUR2009**  **LEVY2015**  **MAHER2011**  **NISSIM2012**  **REED2015**  **REEVE2009**  **REEVE2010**  **SINDING2002**  **WINTERLING2004**  **DE GUZMAN2013**  **FOLEY2006**  **HUBBARD2011**  **TOTAL** | **USA**  **Canada**  **England**  **England**  **Sweden**  **New Zealand**  **USA**  **Norway**  **England**  **Scotland**  **Norway**  **Norway**  **Australia**  **USA**  **USA**  **USA**  **Turkey**  **Turkey**  **Brazil**  **Uganda**  **Thailand**  **England & International**  **England, Australia & USA**  **Wales**  **Wales**  **Canada**  **England**  **Portugal**  **USA**  **Canada**  **Canada**  **China**  **England**  **England**  **USA**  **England**  **Norway**  **Switzerland**  **England**  **England**  **Ireland**  **Australia**  **Canada**  **England**  **USA**  **Germany**  **Sweden**  **Sweden**  **The Netherlands**  **Sweden**  **Norway**  **Denmark**  **Sweden**  **England**  **Scotland**  **Norway**  **Sweden**  **New Zealand**  **England**  **England**  **USA**  **Denmark**  **UK**  **England**  **Canada**  **UK**  **England**  **England**  **Canada**  **Sweden**  **Philippines**  **USA**  **Canada** | **All - Childhood**  **All - Childhood**  **All - Adolescence and young adulthood**  **All - Adolescence and young adulthood**  **All – Adolescence**  **All - Adolescence and young adulthood**  **All - Adolescence and young adulthood**  **Breast & Prostate**  **Breast-women**  **Breast-women**  **Breast-women**  **Breast-women**  **Breast-women**  **Breast-women**  **Breast-women**  **Breast-women**  **Breast-women**  **Breast-women**  **Breast-women**  **Breast-women**  **Breast-women**  **Breast-men**  **Breast-men**  **Breast-men**  **Breast-men**  **Breast-men**  **Gynaecological**  **Gynaecological**  **Ovarian**  **Ovarian**  **Ovarian**  **Cervical**  **Vulva**  **Vulva**  **Penis**  **Colorectal**  **Colorectal**  **Colorectal**  **Multiple myeloma**  **Multiple myeloma**  **Multiple myeloma**  **Multiple myeloma**  **Multiple myeloma**  **Cutaneous T-cell Lymphoma**  **Lymphoma**  **Leukaemia**  **Oesophageal**  **Oral**  **Retinoblastoma**  **Recurrent breast cancer**  **Incurable**  **Incurable**  **Lung**  **Prostate**  **Prostate**  **Advanced prostate**  **Prostate and advanced prostate**  **Terminal**  **Advanced breast cancer**  **Advanced**  **Advanced colorectal**  **Advanced**  **Advanced prostate**  **Incurable multiple myeloma**  **Advanced lung or gastrointestinal**  **Metastatic breast**  **Advanced**  **Terminal**  **Metastatic breast**  **Advanced gastrointestinal**  **Breast or Colorectal**  **All**  **All** | **X**  **X**  **X**  **X**  **X**  **X**  **X**  **X**  **X**  **X**  **X**  **X**  **X**  **X**  **X**  **X**  **X**  **X**  **X**  **X**  **X**  **X**  **X**  **X**  **X**  **X**  **X**  **X**  **X**  **X**  **X**  **X**  **X**  **X**  **X**  **X**  **X**  **X**  **X**  **X**  **X**  **X**  **X**  **X**  **X**  **X**  **X**  **X**  **X**  **X**  **X**  **X**  **X**  **X**  **X**  **X**  **X**  **X**  **X**  **X**  **X**  **X**  **X**  **X**  **X**  **X**  **X**  **X**  **X**  **69** | **X**  **X**  **X**  **X**  **X**  **X**  **X**  **X**  **X**  **X**  **X**  **X**  **X**  **X**  **X**  **X**  **X**  **X**  **X**  **X**  **X**  **X**  **X**  **X**  **X**  **X**  **X**  **X**  **X**  **X**  **X**  **X**  **X**  **X**  **X**  **X**  **X**  **X**  **X**  **X**  **X**  **X**  **X**  **X**  **X**  **X**  **X**  **X**  **X**  **X**  **X**  **X**  **X**  **X**  **X**  **X**  **X**  **X**  **58** | **X**  **X**  **X**  **X**  **X**  **X**  **X**  **X**  **X**  **X**  **X**  **X**  **X**  **X**  **X**  **X**  **X**  **X**  **X**  **X**  **X**  **X**  **X**  **X**  **X**  **25** |

**Online data Supplement 3: Data extraction table**

| # | Study ID | Country | Approach | Method | Participants/Setting | Main Findings | Quality rating |
| --- | --- | --- | --- | --- | --- | --- | --- |
| 1 | PROUTY2006 | USA | Phenomenology | The purpose of this phenomenological study was to examine *the lived experience of adults who survived childhood cancer.* Semi-structured interviews were used to collect data. | Twelve people (between the ages of 19 and 51) participated. Each participant had been diagnosed and treated for cancer before the age of 18 and had been symptom free for at least one year. Potential participants were recruited from a hospital-based clinic where adult survivors of childhood cancer receive follow-up care, and through an advertisement published in a newsletter that is mailed to cancer survivors. | Four themes emerged:   1. Ongoing consequences for having had cancer 2. Living with uncertainty 3. Cancer is embodied or 'a part of who I am' 4. Support is valued. | 13/18  High |
| 2 | WOODGATE 2003 | Canada | Grounded Theory | The aim was to describe *the childhood cancer symptom course experienced by children with cancer from the perspectives of the children and their families.* Open-ended formal interviews and participant observation of children was used.  Participants were observed during various periods in their illness, at various locations, and at different points in time during the study.  Informal interviewing also took place during periods of participant observation.  Children and family data are presented separately. | A theoretical sample of 39 children (4.5–18 years of age) with a variety of cancer diagnoses and their family members participated. 103 interviews were conducted with children during the course of the study. | Symptoms were a very big part of the cancer experience for the children. Five themes specific to symptoms emerged:   1. Short-term pain for long-term gain 2. You never get used to them [symptoms] 3. They [symptoms] all suck 4. It sort of helps [symptom management] 5. They are all the same but they are all different (the children reinforced that every child with cancer experiences the disease and its symptoms in unique ways). | 14/18  High |
| 3 | FERN2013 | England | Participatory research | The study aim was to provide greater insight into *adolescents and young peoples' experience of cancer* (and the care they have received).  Participatory research was used - based on working with Adolescents and Young People (AYP) as co-researchers as well as participants.  Data was collected in the form of a workshop which included semi-structured peer-to-peer interviews.  Thematic analysis was used.  A second ‘workshop’ was held to affirm researcher findings after analysis. | Eleven adolescents and young people (13-25years) who were diagnosed with cancer and treated within the last 5 years participated in a workshop. The same group of participants were invited to a second workshop (to reaffirm researcher findings after analysis). Six participants attended the second workshop.  Four participants were members of a consumer group trained in research methods. | Eight core themes emerged:   1. Life-changing impact of diagnosis: “cancer diagnosis made me grow up”. This theme relates to coming to terms with a cancer diagnosis. 2. Provision of information: “I’m more than my cancer” 3. Place of care: “If I had known…I would have travelled there” 4. Role of health professionals: “Cancer nurse tells mum to get out!!!” 5. Coping: “It’ll finish one day, treatment’s not forever”. This theme describes how participants established a “new normal”. 6. Peers: “Rehab[ilitation] buddies for cancer survivors 7. Psychological support: “Counselling for   patients to cope”   1. Life after cancer: “The tumour’s out, but what now?” This theme describes how a diagnosis of cancer changed participants’ planned life course.   The main limitation is lack of clarity re: aims and findings - overlap between LWBC and experience of cancer care. | 14/18  High |
| 4 | GRINYER2007 | England | Not reported | The study explored *the experience of having cancer in adolescence and young adulthood.* The aims were to understand how age and life stage affect the illness experience and impact upon biographies and life trajectories. The research question was *‘what is it like to be a young adult with cancer?’* The data were gathered primarily through the use of in-depth qualitative interviews, but also through a small number of written narratives. | The adolescents and young adults (n=40) were recruited to the study using convenience sampling. The required characteristics of the proposed sample were only that they had been between the ages of 15 and 25 years when diagnosed with cancer. Participants were recruited predominantly from two hospital Trusts, through a specialist teenage ward in one Trust and through general oncology services in the other. Data were gathered from 20 participants on the specialist ward, from 15 through general oncology services and from five young people who contributed written narratives. Ages at interview ranged from 16 to 26 years. | Three themes emerged:  1) The Disruption of Life Trajectories  Central to the testimonies of the participants was the way in which their life trajectories had been disrupted – with examples of ‘lives interrupted’ at a crucial stage and of significant opportunities that had to be relinquished or postponed. All feared a lasting effect from the enforced change of plans.  2) The Loss of Independence  A significant effect of the illness on the biographies of the participants was their loss of independence and the concomitant disruption to their living arrangements.  3) The Setting of Care  Recruiting from both a specialist ward and a Trust without age-specific facilities allowed some insight into the difference in experience between the differing settings of care. | 9/18  Medium |
| 5 | MATTSSON  2007 | Sweden | Not reported | The study explored *consequences of cancer during adolescence* experienced two years after diagnosis.  Telephone interviews were conducted. Two questions were asked:   1. *What, if anything, is bad for you due to the cancer disease? and* 2. *What, if anything, is good for you due to the cancer disease?*   The answers were analysed using content analysis. | 38 Swedish-speaking adolescents (13–19 years) newly diagnosed with cancer or with a recurrence of cancer treated with chemotherapy, and who were cognitively, emotionally, and physically capable of participating, were recruited, from three Swedish paediatric oncology centres. Those diagnosed with a recurrence were eligible if they had been disease free and off treatment for at least one year. | Four categories of negative experiences were identified:   1. A problematic body (physical and appearance problems and the consequences of these) 2. Unpleasant thoughts and feelings (e.g. with regard to relapse and memories from hospital visits and in connection with questions about the disease) 3. Outside the circle of friends (being isolated or having lost touch with friends) 4. Difficulties with schoolwork (school work takes a lot of effort and energy and extra work is necessary to catch up on missed time at school).   Six categories of positive experiences were identified:   1. A more positive view of life (another view of life. An awareness of death means that problems assume other proportions and that the present and day-to-day life are at the centre of things) 2. Good self-esteem (an inner change with regard to maturity and development into a responsible person with good self-esteem, amongst other things regarding the person’s own body) 3. Knowledge and experience with regard to disease and hospital care (knowledge and experience of disease and a secure relationship with hospital care) 4. Good relations (good ability to understand and thus help other people. Values close relations with friends and other people) 5. Broader perspectives (new leisure-time activities and occupational plans) 6. Material gains (new things, social insurance money). | 11/18  Medium |
| 6 | WICKS2010 | New Zealand | Not reported | In-depth interviews  were conducted to gather information on *Adolescents and young peoples’ experience of cancer diagnosis and treatment.*  Thematic analysis was used to identify and analyse patterns within the text. | Ten young people (six males, four females) participated in the in-depth interviews. Eligible participants were age 14–19 years at the time of diagnosis (variety of cancers), and treated at Wellington Hospital between 1 January 2002 and 31 December 2006. | Six main themes emerged from the analysis:   1. Treatment issues 2. Hospital facilities 3. Information provision 4. Physical effects of treatment 5. Emotional effects of treatment and 6. Impact on developmental processes.   Two strongly recurring sub-themes were identified in the analysis:   1. Loss of control 2. Benefit finding (positive aspects to being treated for cancer) | 12/18  Medium |
| 7 | JONES2010 | USA | Phenomenology | The purpose of the study was to conduct interviews with Latino adolescent cancer survivors. A hermeneutic phenomenological approach was selected based on the focus on *experiences and meanings of Latino adolescents’ cancer survivorship.* In-depth interviews were conducted with participants. Using a phenomenological approach to interviews, the subjects were asked five open-ended questions about the experience and meaning of surviving cancer. | Participants were: Latino Adolescents and young people between the ages of 14–21 who were all post treatment (n=9). Purposive sampling was used to identify participants who met the inclusion criteria: a cancer diagnosis, between the ages of 12–21 years old, post treatment, self-identified as Latino, and English speaking. | Seven themes emerged:   1. Gratitude 2. humour/positive attitude 3. empathy for younger children with cancer 4. god and faith 5. cancer happens for a reason/cancer changed my life 6. familial support, 7. staff relationships. | 11/18  Medium |
| 8 | GRIMSBO  2011 | Norway | Not reported | The aim of the study was to explore the use and content of patients’ online communication to oncology nurses to gain a ‘snapshot’ of *breast and prostate* *patients’ experiences of living with cancer.*  Content analysis was used. | 276 email messages from 38 breast and 22 prostate cancer patients were used. The participants were part of a wider RCT (randomly assigned to the experimental group) which tested an internet-based support system. | Four main themes emerged:   1. Living with physical symptoms and problems: “Is this a side effect of treatment or is it ‘just me’?” 2. Living with the fear of relapse: “I walk around with my nerves on edge, terrified of the slightest sign of pain, no matter where it might arise” 3. Concerns for everyday life: “I don’t want to do anything wrong” 4. Unmet information needs from healthcare providers: “I have a feeling of hanging in the air” | 15/18  High |
| 9 | TRUSSON  2016 | England | Narrative | Individual interviews were conducted to explore *the experience of breast cancer.* | Twenty-four women (aged between 42 and 80) who had been treated for early stage breast cancer in the UK between 6 months and 29 years previously, were recruited through local media. | Five themes were identified:  1. Biographical disruption and liminality  2. Fear of recurrence  3. Embodied reminders  4. Relationships  5. Changes in outlook  Although no longer ill, an  ongoing fear of recurrence combined with embodied changes prevented a return to ‘normal’ i.e. a pre-cancer state in terms of health status, identity and relationships. The authors argue that following the biographical disruption of breast cancer, a ‘new normal’ entails a continual renegotiation of identities, daily lives and futures as time passes and lives evolve. | 14/18  High |
| 10 | McCANN2010 | Scotland | Grounded theory | The aim of the study was to *explore the experiences of people with breast cancer within the first year following diagnosis.* Longitudinal interviews were conducted at three time-points within the first year following diagnosis with 12 women with breast cancer. | Specific data are drawn from 36 in-depth interviews with 12 women with breast cancer in Scotland. Taken as a whole, the study included 66 patients with cancer. | Two key concepts emerged from the data:  1. Identity Transition: moving between health and illness  2. Making the Transition to the Future? Living with breast cancer and moving on  Identity transition emerged as a result of the changes and  adaptations participants were required to make as a result of their diagnosis. Making the transition to the future emerged as a result of the challenges these women faced living with cancer. | 10/18 Medium |
| 11 | LANDMARK  2001 | Norway | Grounded theory | This study aimed to *describe how women diagnosed with breast cancer experienced living with the disease.* In-depth open-ended interviews were conducted.  Data were analysed using grounded theory. | The sample consisted of 10 Norwegian women selected from a group of patients with newly diagnosed breast cancer, admitted  to an out-patient oncology clinic during the period  August–December 1997. | Data revealed that existential awareness was a central phenomenon in the women’s experience. This central finding created the basis for the core category in data: the will to live. This core category includes existential aspects such as   1. different levels of life expectations 2. the fight against death 3. life related to the future 4. religious beliefs and doubts 5. increased awareness   of values in life. | 15/18  High |
| 12 | LANDMARK  2002 | Norway | Grounded theory | The aim of this qualitative study was to *describe the experience of living with breast cancer.* In-depth open-ended interviews were conducted.  Data were analysed using grounded theory.  Extended analysis of LANDMARK2001 | The sample consisted of 10 Norwegian women (aged 39-69) selected from  a group of patients with newly diagnosed breast cancer,  admitted to an out-patient oncology clinic during the period  August–December 1997. The following criteria were used to select participants: diagnosis of breast cancer under primary  treatment; women over 20 years of age; patients without distant metastases. | Results indicated that living with newly diagnosed breast cancer included different experiences related to physical, psychological, social and existential issues. The existential life sphere emerged as a main domain. The will to live emerged as the central theme in this domain in relation to how the women experienced living with breast cancer.  Six categories related to the will to live:  1. Unknown strength – experiences related  to the will to live  2. Emotional chaos – experiences related to emotional reactions  3. The body broken and torn – experiences related to  bodily, physical change  4. Loss of breast – experiences related to female identity  5. Doing something else – experiences connected  to meaningful activities  6. For better and worse – experiences related to social  support | 13/18 High |
| 13 | OXLAD2008 | Australia | Not reported | Focus groups were conducted to *explore experiences of living with breast cancer.* | Ten women aged 36–68 years who had recently completed treatment for early-stage breast cancer at a South  Australian public hospital took part in one of three focus groups. | Participants reported a sense of apprehension about the future at the completion of primary treatment.  In addition to this, five specific areas of concern were identified including   1. Physical sequelae of treatment 2. Intimacy issues 3. Fear of recurrence 4. Benefit finding 5. Optimism versus pessimism about the future. | 13/18  High |
| 14 | BOEHMKE  2006 | USA | Phenomenology | To *explore shared meanings and common experiences for women following a diagnosis of breast cancer.*  Secondary analysis of interview transcripts was undertaken using the 7-stage hermeneutic process. | The study used purposive, convenience sampling and recruited 30 women. | Four themes emerged:   1. Changing health overnight 2. Erasing of a former self 3. Appraising of illness 4. Approaching the future-now what?   The constitutive pattern was transitioning from health to illness. | 14/18 High |
| 15 | FAKHRI OBEIDAT2012 | USA | Phenomenology | The objective of the study was to *understand the experience of being diagnosed with and undergoing surgical treatment for early-stage breast cancer* among Arab American women. Data were collected using individual interviews and analysed using the Heideggerian hermeneutical  methodology. | Ten Arab American women who were surgically treated for early-stage breast cancer (stages 0, I, II, IIIA) in the northeast and Midwestern United States were recruited using snowballing and networking sampling techniques. Participants were included if they were older than 21 years, spoke either Arabic or English, and were mentally and physically able to consent to and participate in the study. | Five themes emerged:   1. Breast cancer as a life-threatening illness   Sub-themes:  Breast cancer means death  Reacting emotionally  Mastectomy for peace of mind   1. Breast cancer as a fate   Sub-themes:  Putting breast cancer diagnosis in God’s hands  Testing faith with the challenge  Collaborating with God for coping   1. Seeking information about own diagnosis and treatment   Sub-themes:  Understanding details and rationale for treatment  Seeking information beyond that provided   1. Trusting physicians for treatment choice and/or decisions   Sub-themes:  Participating in treatment decision making  Preferences for choice/no choice and role in decision making  Preferences for surgeons’ gender and/or religion   1. Accessing/finding social support & Family, friends, and neighbors support   Sub-themes:  Husbands’ support  Distance from families overseas  Healthcare providers’ support  A constitutive pattern emerged: Confronting breast cancer as a challenge from God. | 16/18  High |
| 16 | SHERMAN  2012 | USA | Grounded theory | The aim of this grounded theory study was to develop a theory of the process of breast cancer survivorship.  Individual interviews were used. | The purposive sample included 15 women who were diagnosed with early-stage breast cancer, were aged 18 years or older, spoke English, completed treatment (chemotherapy or radiation therapy) ranging from within one year to five years and beyond treatment, and who had no history of breast cancer recurrence or major co-morbid health conditions. | The core category was Reclaiming Life on One’s Own Terms.  Reclaiming Life captures the idea that participants were able to focus beyond illness, thoughts of death, and loss of life as they knew it, to refocus on living life with new perspectives and in ways that promote physical, emotional, and spiritual well-being, on their own terms.  **A process marked and shaped by time**  1. The turning point: breast cancer diagnosis and treatment as a stimulus for change  2. Breast cancer as a part of life  3. Learning to live with breast cancer  4. Creating a new life after breast cancer  **A process influenced by the perceptions of support:**  1. Coming to Terms With Threats and Fears Associated With Diagnosis and Treatment  2. Assuming an Active Role in Self-Healing  3. Gaining a New Perspective  and Reconciling Paradoxes  4. Creating a New Mindset  and Moving to a New Normal  5. Developing a New Way of Being in the World on One’s Own Terms  6. Experiencing Growth Through Adversity  7. Beyond Survivorship  Paradoxical Narratives of Breast Cancer Survivors:   1. Being a Victim of Cancer 2. Being a Survivor of Cancer 3. Cancer as a Part of Me 4. Cancer Is Not Me 5. Cancer as Past 6. Changes are Caused by Cancer 7. Changes Are Not Caused by Cancer 8. Concealing the Diagnosis 9. Revealing the Diagnosis 10. Continuing Fear 11. Letting Go of Fear 12. Creating Boundaries 13. Breaking Boundaries 14. Fear of Death 15. Confronting Death 16. Feeling Different 17. Not Feeling Different 18. Suffering From Uncertainty 19. Living With Uncertainty 20. Feeling Vulnerable 21. Feeling Stronger | 14/18  High |
| 17 | KUCUKKAYA  2010 | Turkey | Not reported | The aim was to explore the nature and degree of *positive life changes reported by women living with breast cancer.* Open-ended questionnaires were used to collect the data. Content analysis was used to analyse the qualitative data. | The participants were 84 women aged between 30 and 60 years, who were diagnosed with breast cancer (Stages I or II) at Hacettepe University Oncology Hospital, who are at least elementary school graduates, and who have not received any  psychiatric diagnosis or assistance from a mental health professional  before. | Four main themes emerged:   1. changes in self-perception   Sub-themes:  Increased self-awareness and acceptance of old and  renewed personality  Increased appreciation of personal worth   1. empowerment   Sub-themes:  Improvement in personality  Insight concerning how to avoid stress and conflict  Becoming more assertive  Tolerance  Changes in lifestyle  Changes in perception of cancer and medical issues   1. greater appreciation of life   Sub-themes:  Positive changes in world view  Changes in life priorities and goals  Renewed recognition of life as a second chance   1. changes in interpersonal relations   Sub-themes:  Better relationships with loved ones  An increased sense of connection with others  An increased sensitiveness towards others’ feelings  Greater willingness to help sick people | 8/18  Medium |
| 18 | CEBECI2012 | Turkey | Not reported | The aim was to explore the *experience of women living with breast cancer.*  The data were collected using semi-structured, in-depth individual interviews. | The study was conducted among 8 women with ongoing breast cancer chemotherapy treatment, which occurred in an ambulatory unit.  Purposive sampling was used based on the inclusion criteria: 1) ability to speak Turkish, understand questions, and express one’s  own opinions; 2) diagnosis of breast cancer for at least one year;  3) lack of metastasis or another type of cancer; 4) married status;  and 5) absence of psychiatric disease (or history of psychiatric  disease). | Three major themes were identified:   1. needs (the need for spouse and family support, the need to worship, and the need to receive and share information) 2. living with losses (loss of the breast and of one’s hair) 3. changes (changes in one’s normal life, change in self-perception, changes in the perception of the   value of health, and a  greater appreciation for  life). | 11/18  Medium |
| 19 | Da COSTA VARGENS  2007 | Brazil | Phenomenology | Interviews were conducted to explore *the lived experiences of women receiving treatment for breast cancer.* Data were interpreted through Heidegger’s existential phenomenology. | Participants were: 11 women diagnosed and treated for breast cancer. Purposive sampling was used. Selection criteria was that the women (a) were over the age of 18 years and (b) were diagnosed with breast cancer during the period of 1993 to 2001. | Four themes were identified:   1. gaining a positive attitude for life 2. wanting to be recognized as a woman with certain needs 3. considering body image/self-image 4. making efforts to hide. | 10/18  Medium |
| 20 | GONZAGA  2013 | Uganda | Not reported | The purpose of this study was to *explore the lived experiences of women diagnosed and living with breast cancer.*  In-depth individual interviews were conducted and findings were summarised into themes. | This was an exploratory qualitative study using a convenience purposive sample (n = 12) of women confirmed with breast cancer and reporting to the Radiology department for imaging. | Four major themes summarising the womens' experiences included:   1. Thought of Death 2. Strength to live 3. Loss of female identity 4. sexuality and Coping mechanisms. | 6/10  Low |
| 21 | LIAMPUTTONG2016 | Thailand | Feminist framework | This study explored *the lived experiences and meaning-making of breast cancer* among women in southern Thailand.  In-depth interviews with 20 women living with breast cancer were conducted and invited to take part in a drawing method. The drawing method constitutes a form of visual imagery and is a contemporary method used to collect information from vulnerable people and for sensitive topics. The research is situated within the feminist framework. | The participants of this study were: 20 Thai women  who had been diagnosed with breast cancer in Southern Thailand. The purposive sampling method was used to select key informants who met the study criteria and could provide rich data relevant to the aim of our study. | Four themes emerged:   1. Breast cancer means death 2. Meaning-making: perceived causes of breast cancer 3. Meaning-making: dealing with breast cancer 4. living for children 5. accepting fate and karma 6. Religious beliefs and practices as support | 12/18  Medium |
| 22 | DONOVAN2007 | England & international | Phenomenology | The research aim was to explore *the lived experience of breast cancer for men.* The study  adopted a henomenologic framework. Data were obtained from in-depth, semi-structured interviews with 5 participants from the United Kingdom. Ten (n = 10) self-volunteered  participants from overseas contributed contextual data via  e-mail correspondence. Analysis proceeded according van Manen’s approach and was further contextualised within the 4 fundamental  existential themes of spatiality, corporeality, temporality, and relationality. | Participants were: men with breast cancer (n=5).  Ten (n = 10) self-volunteered  participants from overseas contributed contextual data via  e-mail correspondence. | 4 key themes emerged:   1. Living with male breast cancer 2. Concealment as a strategy for managing the diagnosis 3. A contested masculinity 4. Interacting with health services | 6/18  Low |
| 23 | NAYMARK2006 | England, Australia & USA | Not reported | In depth interviews were conducted over a 4 month period to discuss *experiences of living with male breast cancer.* Thematic analysis was used to analyse the data. | Purposive sampling was used to recruit 6 men diagnosed and treated for breast cancer (recruited from three countries) and 5 Australian health professionals. Results from men with breast cancer and health professionals are reported separately. | Findings highlighted the gender disparities within breast cancer research, treatment and services (emphasis on diagnosis and support needs).  Men related feeling shocked and foolish upon being diagnosed with a cancer they once conceived as a solely female concern. One man claimed that he had no previous knowledge of male breast cancer and, in spite of his treatment for breast cancer, denies having gained any understanding of it. Initially the men found that their diagnosis generated much bewilderment and uncertainty among friends and family as to how to display support and understanding.  Men also reported stories of considerable dissatisfaction  with support services and information following their mastectomies. | 6/18  Low |
| 24 | FRANCE 2000 | Wales | Phenomenology | The aim was to explore *the lived experience of male breast cancer.* In depth interviews were conducted within a phenomenological framework. Content analysis was used for data analysis. | 6 men who had completed radiotherapy or chemotherapy treatment for breast cancer participated. | 7 categories emerged:  1. Delay in the diagnosis  2. Shock - a reaction to the diagnosis  3. Stigma - attitudes to male breast cancer  4. Body image  5. Causal factors (environmental toxins, familial)  6. Provision of information (relating to the condition and to the treatment)  7. Emotional support and counselling | 7/18  Medium |
| 25 | WILLIAMS2003 | Wales | Not reported | The aim of the study is to explore *the experiences of men who have had breast cancer*, and to compare the experiences to those of women who have had breast cancer and health professionals.  Four focus groups were conducted: 2 groups with men who have breast cancer, 1 group with women who have breast cancer and 1 group with health professionals. Thematic analysis was used for analysis. | Participants were: men with breast cancer, women with breast cancer and health professionals (n=27 participants in total). Findings of each stakeholder group are reported separately. | Four themes were identified:  1. Diagnosis  2. Disclosure  3. Support  4. Gender-specific information | 6/18  Low |
| 26 | PITUSKIN2007 | Canada | Not reported | The objective of the study was to *describe the experiences of Canadian men diagnosed with breast cancer.* Unstructured interviews were conducted with  20 men. | A self-selecting sample of 20 men with breast cancer were recruited via the Alberta cancer board cancer registry. | Participants experienced concerns related to the lack of awareness of male breast cancer within both public and health professional groups. Many men suffered stress related to the cancer diagnosis, body image concerns and role strain. The lack of male-specific breast cancer information was identified as a major concern. All denied interest in traditional support groups. In retrospect, a number of men felt the breast cancer experience vastly improved their lives.   1. Breast cancer diagnosis: knew men could get breast cancer, problem getting attention from doctor 2. Disclosure: Some men described reluctance   to share their unusual  problem: Others felt  that improving  awareness of male  breast cancer was an  important duty.   1. Coping: While some men described debilitating emotional   stress related to the  diagnosis, other men  experienced breast  cancer as a brief  medical event without  major long-term  ramifications.   1. support: married participants   reported relying on  their wives as their  sole source of support.  None of the 20 men  expressed interest in  attending formal  support groups before,  during or after their  treatments.  Within the interviews, none of the participants provided specific recommendations regarding supports they felt should be available for men with breast cancer. However, a small number of men felt that it would have been helpful to speak to another man who had faced the same concerns and decisions.   1. Lack of information: consistent lack of medical information about treatment recommendations. 2. Body image: The permanent stigma of the mastectomy scar 3. A new life: Younger men reported that while their breast cancer experience was   difficult to endure at  the time, their lives  had been vastly  improved.  These men spoke of new insight, changed views and different priorities in life. | 8/18  Medium |
| 27 | ROBERTS2009 | England | Grounded theory | The aim of the study was to develop an understanding of the experience of a gynaecological cancer diagnosis on women. Interviews were conducted using grounded theory. | Participants were:  20 women who were  at least 12 months post-surgical treatment for gynaecological cancer. | The findings highlight the degree of biographical disruption that occurs following illness.  Two key themes emerged:  1. The search for meaning: exploring the influence of emergence  2. Living with risk: reclaiming the past for future orientation | 16/18  High |
| 28 | LARANJEIRA  2015 | Portugal | Phenomenology | The aim of the study was to *understand the temporal existential experiences of women who are living with gynaecological cancer.* Interviews were conducted using a phenomenological-hermeneutical,  reflective life world and feminist perspective. | Participants who had received treatment for gynaecological cancer (uterine, cervical and ovarian) and were now in complete recovery took part (n=10). | A main metaphorical meaning was identified: Outside looking in.  This meaning had three constituents:  1. Personal experience - cancer legacy  2. Biographical disruption - feeling 'off time', realising mortality  3. Refiguring the life world - knowing the time (existential: lived time) | 14/18  High |
| 29 | THOMPSON  2007 | USA | Grounded theory | The study explored *the subjectivity of nine women living with stage-three ovarian cancer.*  Data collection involved two semi-structured  interviews followed by the collection of a sample of reflective writing  Grounded theory methods were used to analyse data. | 9 women living with stage 3 ovarian Cancer (recruited via two support groups) participated. | The recurrent nature of the disease with its poor survival rate was found to instill a kind of sustained trauma that is accentuated by experiences of suffering in connection to loss.  **Communicative Alienation**  Difficulty communicating one’s experience is a defining feature of liminality. The participants of this study described *two* forms of communicative alienation: an inability to articulate  their experience due to an unavailable other with whom to share their subjectivity,  and second, an inability to find words during the phase of treatment for the disease  (ovarian cancer most often being a disease of multiple treatment episodes with  periods of subjective wellness in-between).  **Boundedness**  A powerful theme that repeated itself in these women’s illness narratives  involved an awareness of limited available time. The majority of the sample expressed that not only was the future limited, or “had changed,” but that the experience of time *itself* was no longer the same. | 12/18  Medium |
| 30 | BOWES2002 | Canada | Grounded theory | The aim of the study was to explore *the emotion of anger in women living with ovarian cancer and dealing with an early death.* However, early analysis revealed that anger was not a core issue. Consequently, the women’s psychological experience of living with ovarian cancer was explored. Semi-structured interviews were conducted using grounded theory. | A convenience sample of 9 women who had completed chemotherapy treatment for ovarian cancer participated. | The findings are specific to the concerns of the women regarding ‘dealing with an early death.’ The core category to emerge was: Finding meaning in life (which involved self-reflection, reappraisal of life and development of new short-term life goals)  Sub categories:  1. Hope  2. Physical wellness  3. Action strategies included: sharing experiences, family support, searching for meaningful information, religion, rationalising  4. Interactional strategies: crying, humour, anger  5. perceived wellbeing/despair | 9/18  Medium |
| 31 | BURLES2013 | Canada | Phenomenology | This research explores *women’s lived experiences of ovarian cancer* and how they negotiate and make sense of illness-related issues.  Face to face interviews and email follow-up informed by hermeneutic and social phenomenological approaches were conducted. | 16 participants with Ovarian Cancer (whose ages ranged from37 to 68 years, with a mean age of 52 years). All participants were diagnosed when younger than 60 years, with time since diagnosis ranging from 9 months to 13 years. The stage at diagnosis varied among participants, with some having early-stage and others having advanced ovarian cancer. | 5 broad themes related to an overall category of 'cautious optimism' emerged.   1. ‘‘A Whole Different Lifestyle’’: Altered Health Status and Body 2. ‘‘Wow, I’m Really This Sick and I Can’t Do   Anything’’: Disruptions  to Everyday Life   1. ‘‘One Never Has Cancer ‘Alone’’’: Shifts   In Relationships   1. ‘I Still Wonder About Tomorrow’’:   Uncertainty of the  Future   1. ‘It’s Made Really Big Changes in How I See a   Lot of Things’’: Coping  With and Finding  Meaning in Illness  The essence reflects participants’ ongoing negotiation of the implications and potential outcomes of ovarian cancer and their efforts to be hopeful yet realistic given the uncertainty they face. Despite their unique  experiences, participants shared the experiences of changes in health status and the body, disruptions to everyday life and relationships, and the uncertainty of their future. Developing coping strategies and finding meaning in ovarian cancer allowed them to adapt to these and other ongoing physical, psychological, and  social issues. Participants across the illness trajectory shared a sense of cautious optimism in their oscillations between uncertainty and hope as they sought to attain a sense of normality in their lives. | 13/18 High |
| 32 | DING2015 | China | Phenomenology | The aim of this study was to describe *the experiences of Chinese women with cervical cancer* in the first 3 months after diagnosis. A descriptive phenomenological method was used. Fourteen  participants were interviewed at the time of diagnosis and 10 were reinterviewed  3 months later. | The participants’  ages ranged from 30 to 54 years. All were in the early  stages of cervical cancer (n=14). | Five themes were discerned: 1. Dealing with the emotional chaos down to the hassles caused by the disease and its treatment  2. Handling the diagnosis and the disease in my own way and in accordance with Chinese culture  3. Instinctively depending on my partner and experiencing improved relationships within the family but no sexual life  4. Sharing information and support with fellow patients but hiding the diagnosis from friends and acquaintances  5. Returning to previous existence wanting to be treated as normal but with changed attitudes to life. | 14/18 High |
| 33 | JEFFERIES2009 | England | Phenomenology | The aims of the study were to explore *the lived Experience of Women With Cancer of the Vulva.*  Using interpretive phenomenology, interviews were conducted.  The data were analysed using framework analysis. | Thirteen women were interviewed. The women were all younger than 50 years, which reflected the increased incidence of vulval cancer in the younger age group. Four of the women had precancerous conditions previously, whereas the other 9 women had no prior vulval tissue abnormality. The women were interviewed once between 6 months and 5 years following their surgery. | Key to the findings is the emergence of the concept of searching, the subject of this article.  Searching is characterized  by a search for control and, for example, considering  the physical components, searching to control the distress. It was related to the women’s own perception of their symptoms, and their journey toward a definitive diagnosis and treatment.  The concept of searching also has an existential meaning for  the women at the time of their diagnosis of cancer, as they  adjust to the implications of a potentially life-threatening illness on them, their family, and their future.  To summarize, the concept of searching consists of:  searching to control  searching for meaning  searching: the time element | 9/18 Medium |
| 34 | JEFFERIES 2012 | England | Phenomenology | The aims of the study were to explore *the lived Experience of Women With Cancer of the Vulva who had undergone surgical treatment.*    The data were analysed using framework analysis.  Extended analysis of JEFFERIES2009. | This study describes an exploration of the lived experience of 13 British women with cancer of the vulva who underwent surgical treatment. The women, younger than 50 years, were identified by  purposive sampling and interviewed between 6 months and 5 years after surgery. | The women’s lived  experience is described in its entirety by the concept of invisibility, characterized as  something ‘‘no one can see,’’ ‘‘heard of,’’ and ‘‘talks about.’’  The impact of this invisibility is discussed as the ‘‘invisibility of understanding’’ and  ‘‘invisibility of support.’’ | 7/18  Medium |
| 35 | GORDON2017 | USA | Phenomenology | This study examined the *experiences of men with penis cancer.* Face-to-face semi-structured interviews were conducted and  Heidegger’s hermeneutic phenomenology served as the philosophical  Underpinning. Colazzi’s method was used to analyse the data. | A purposive sample of 13 men who had been treated for penile cancer was recruited. | Six main themes emerged from the data:   1. Misdiagnosis   Sub-themes:  Self-discovery and report of symptoms  Delayed diagnosis  Dissatisfaction   1. Secrecy   Sub-themes:  Reluctance to disclose diagnosis  Stigma and embarrassment  Surviving penis cancer   1. ‘‘Cancer is cancer,’’   Sub-themes:  Cancer is greater than the penis  Emotional turmoil  Surprise/shock  Anxiety  Anger  Scared and embarrassed  Fear  Denial  Relief  Acceptance of cancer diagnosis  Urination and side effects   1. Sexual Issues   Sub-themes:  Ruins sex life  Challenges to manhood  Avoidance/abstinence  Causes marital problems  Lack of professional help with changes in sexual life  Sex activity not as much a concern with older participants   1. Support 2. Awareness and Prevention.   Sub-themes:  Lack of knowledge about penile cancer  Seek early treatment  Circumcision | 13/18  High |
| 36 | APPLETON2013 | England | Phenomenology | The aim of the study was to *explore experiences of living beyond colorectal cancer.*  Semi-structured, face to face interviews and interpretative phenomenological analysis (IPA) was used. | A purposive sample of thirteen participants who had completed a primary course of curative treatment for colorectal cancer within the past 6 months to 5 years, with no further treatment pending  were recruited. | Three super-ordinate themes were generated:   1. Partnership   with the multidisciplinary team   1. Enablers 2. The self beyond cancer.   These themes are not linear but can be described as  a dynamic process that occurred for each person at different points along their pathway. | 13/18  High |
| 37 | SALAMONSEN 2016 | Norway | Not reported | The concept of “patient pathways” in cancer care is most commonly understood as clinical pathways, operationalised as standardized packages of health care based on guidelines for the condition in question. In this understanding, patient pathways do not address multimorbidity or patient experiences and preferences. This study *explored patient pathways understood as the individual and cultural life course,* which includes both life and health events.  A qualitative, open-ended research design (interviews and workshop) was chosen to explore subjec­tive and experience-based patient perspectives. | Nine Norwegian patients recently diagnosed with rectal cancer Tumor-Node-Metastasis stage I–III participated in qualitative interviews, five times over 1 year. Five patients later participated in a workshop where they made illustrations of and discussed patient pathways. | Findings identified “disrupted life courses” and a disruption in patient pathways  **Disruptive events related to the cancer diagnosis and life with cancer**   1. Before diagnosis: the illness and death of significant others   **Why disruptive?**  Emotional burdens: loss, grief and mourning, shock, anxiety, anger, uncertainty, guilt, unwanted changes, less able to care for other family members, depression  Practical burdens: economy, job combined with home care and hospital visits, housekeeping, moving   1. At point of cancer diagnosis   **Why disruptive?**  Shock, trauma, anxiety, uncertainty, grief over “the lost, expected life”   1. After diagnosis: disruptive health events   **Why disruptive?**  Uncertainty, lack of food, lack of care, bad prognosis, difficult visits to the hospital.  Diarrhea and stoma disrupt social life, work life, and sex life, Fatigue, depression, anxiety   1. After diagnosis: being an ill parent and partner   **Why disruptive?**  Uncertainty, guilt, grief, depression. Negative change in sexual identity  Practical and emotional issues related to the stoma  Guilt, Grief for the disrupted marriage and sexual relationship   1. After diagnosis: not being able to work   **Why disruptive?**  Grief for the loss of network and identity. | 13/18 High |
| 38 | SHAHA2003 | Switzerland | Phenomenology | The study aim was to describe *the experience of living with a diagnosis of colorectal cancer* and what this means to an individual. A phenomenological  study was undertaken based on Heidegger’s philosophy. Longitudinal interviews were conducted and data were analysed by following Colaizzi’s eight-step process. | Seven patients diagnosed with colorectal cancer were interviewed  over a time-span of 13 months (the shortest time frame of interviewing spanned a 6-month period and the longest period was 13 months). | Analysis identified one main category: ‘The Omnipresence of Cancer’  and two sub-categories:   1. ‘Towards Authentic Dasein’ (Towards Authentic Being) 2. ‘Mapping out the Future’   The research demonstrated that having received a diagnosis of cancer means an individual is faced with the potential of lifelong illness and death, feels stigmatised by the diagnosis and is classified as belonging to an illness group. | 10/18  Medium |
| 39 | MOLASSIOTIS2011 | England | Grounded Theory | The purpose of this study was to explore *the*  *experience of living with myeloma* after completion of treatment.  Semi-structured interviews were carried out with 20 patients living with myeloma and 16 of their informal caregivers.  Patient and caregiver data were analysed separately. | Twenty patients living with multiple myeloma were interviewed.  There were 12 female and eight male participants with a mean age of 61.8 years. | The key theme was: ‘coping with and managing myeloma and its consequences’. Categories within this  broad theme included:   1. current and future concerns 2. effects of myeloma in daily life 3. practical, functional and emotional   coping   1. unmet needs. | 12/18  Medium |
| 40 | POTRATA2011 | England | Grounded theory | The aim of this study was to gain greater *insight into the symptoms and distressing experiences of patients living with myeloma.*  A grounded theory study was conducted based on semi structured interviews  [subset of a larger research project] | 15 Patients were recruited using purposeful sampling (paying attention to age and ethnicity) | Five themes emerged:   1. Distress from experienced symptoms 2. Distress from body image changes 3. Distress caused by family and friends 4. Distress from myeloma-related information 5. Distress from stem cell transplantation   Key findings indicate that:   1. Many individual   symptoms were  not considered as  particularly  distressing beyond  the acute phase of  the disease and its  treatment, except  when they  occurred in a  context that was  threatening to the  patients;   1. visible symptoms that showed to other people one’s disease condition may be particularly   distressing,   1. the conditioning phase of the transplant was particularly stressful and a violation to one’s body, described by some patients as ‘being somehow dead’. | 14/18 High |
| 41 | KELLY2011 | Ireland | Phenomenology | The study aim was to explore patients’ *lived experience of being diagnosed with myeloma.*  A hermeneutic phenomenological approach was adopted to analyse data from interviews with 11 patients diagnosed with myeloma. | Eleven participants attending a regional specialist haematology oncology centre, who had been diagnosed with myeloma for more than six months (inclusion criteria) participated. | Four inter-related themes emerged:   1. Lived body: a changed body   Sub-themes:   - Alopecia - Fatigue  1. Lived space: living in limbo   Sub-themes:   - Living with an ‘unknown cancer’ - Stigma of cancer - Feeling ‘lucky’  1. Lived time: time is precious   Sub-themes:   - Fear of recurrence - Limited time with healthcare professionals  1. Lived relations: significance of support   Sub-themes:   - Family support - Protecting others | 8/18  Medium |
| 42 | STEPHENS2014 | Australia | Grounded theory | The aim of the study was to explore *the experiences of long-term survivors of multiple myeloma.*  A series of 47 in-depth interviews were conducted at 6–12 month intervals over 18 months. Interviews were analysed using a grounded theory approach. | 10 long-term survivors of myeloma and their primary support person participated.  Findings from each stakeholder are reported separately. | The creation of ‘illness work’ subsequent to diagnosis. This can be subdivided in 2:   - Risk Work - Emotion Work   To adapt to the effects of both the disease and ongoing medical treatments, participants undertook extensive ‘illness work’. Most of this work fell into two broad categories. Risk work aimed to mitigate risks to the wellbeing  of both the person with myeloma and his/her carer. Emotion work aimed  to manage the feelings of self and others in a protracted cycle of remission and  relapse. | 16/18 High |
| 43 | VLOSSAK2008 | Canada | Not reported | The aim of the study was to gain an understanding of *the impact of multiple myeloma on the patient and family.*  In-depth interviews (conducted face to face (n=1) or by telephone (n=19) were undertaken. Thematic analysis was used for analysis. | This study was conducted with a convenience sample  generated from the list of patients receiving treatment for multiple myeloma from two  haematologists in a large regional cancer centre in Ontario. | Ten themes emerged:  1: The diagnosis was shocking and unexpected  2: There are few options for treatment  3: Worry about the family and how they will handle the diagnosis.  4: Treatment is difficult, long, and very complex  5: Fatigue can be overwhelming  6: Loss of independence  7: Change in self-concept/self-image  8: Obsession on how and when the end will come  9: Fear of recurrence  10: Rationalization of changes in their hopes for the future. | 7/18  Medium |
| 44 | BEYNON2015 | England | Not reported | The aim of the study was to understand in greater depth patients’ experiences of living with cutaneous T-cell lymphoma (CTCL).  Semi-structured interviews were conducted. | A purposive sample of patients with CTCL who attended an inner-city teaching hospital participated (n=19). | Two main themes emerged:   1. Issues regarding diagnosis particularly perceived late diagnosis 2. Impact of the disease on patients’ lives Subthemes included physical well being, practical concerns, and psychological and social wellbeing and coping. | 13/18 High |
| 45 | LEAL2015 | USA | Framework | The aim of this study was to explore patients’ ongoing experiences of living with lymphoma and the changes encountered in this experience over time.  Written reflections to three open-ended questions collected from 28 patients on their experience of cancer at two time points were analysed.  Content analysis using a framework approach was employed to code, categorise, and summarise data into a thematic framework. | Patients with lymphoma who were receiving or had received  treatment within the past 12 months were recruited  from the MD Anderson Cancer Center (n=28). | Framework theme: living with paradox  Four inter related themes   1. Sources of paradox: 2. positive experience 3. negative experience 4. Experience of paradox: 5. coexistence  - contradictory - surreal  1. Resolution of paradox:   i) meaning-making   - Spirituality - Taking control - life perspectives  1. Challenges with medical culture/treatment:   i) foreignness,   1. support   The primary theme concerned moving through a dualistic and complex cancer experience of concurrently negative and positive emotional states across the course of cancer. | 13/18  High |
| 46 | KOENIGSMANN2006 | Germany | Grounded Theory | This study explored the individual’s *reactions to the vital threat imposed by leukaemia.* Semi-structured in-depth interviews were conducted with 12 patients. | 12 consecutive adult patients hospitalised for acute leukaemia within the first week of diagnosis. | 3.1. Illness perceptions  3.1.1. Pre diagnostic complaints  3.1.2. Diagnostic procedures perceived as measurements  3.1.3 Diagnostic procedures as threat  3.1.4 Communication of the diagnosis perceived as overwhelming  3.1.5 Treatment as threat  3.1.6 Age-dependent perception of side effects  3.2. Lay theories and health literacy  3.2.1. Explanation of the symptoms  3.2.2. Possible causes: disorientation and helplessness  3.2.3. Understanding of the disease  3.3. Coping strategies  3.3.1. The role of trust  3.3.2. Possibilities of personal influence  3.3.3. Dealing with the fear of death  3.3.4. Expression of hope | 12/18  Medium |
| 47 | ANDREASSEN2006 | Sweden |  | The aim of this study is to describe patients’ experiences of living with  oesophageal cancer and how they seek information.  Data were collected by semi-structured, qualitative interviews with 13 participants. Content analysis was used to analyse data. | Participants were women and men of different ages who had undergone different treatments for oesophageal cancer. | Four themes were identified: (i) Experiences of becoming a patient diagnosed  with oesophageal cancer is distinguished by the participants’ experiences  of vague symptoms, of receiving the diagnosis and of existential concerns evoked  by the illness.  (ii) Experiences of undergoing investigations and treatment consist of the participants’ experiences of extreme tiredness in relation to investigations and  treatment.  (iii) Experiences of intrusions in daily life is conceptualized by the participants’  experiences of how the illness influenced their daily life.  (iv) Managing a  life-threatening illness consists of a variety of strategies, which the participants  employed to manage their life-threatening illness. | 12/18  Medium |
| 48 | ROING2009 | Sweden | Phenomenology | A hermeneutic  research approach was used to understand, explain, and interpret the transcribed interviews and showed how the  consequences of oral cancer affected the being-in-the-world of the participant | Five patients with oral cancer were interviewed a median time of 4 years after the beginning of treatment. | Three key themes emerged:   1. Existing as oneself 2. Existing in the eyes of others 3. Existing with others | 15/18 High |
| 49 | VAN DIIJK2010 | The Netherlands | Not reported | Semi-structured interviews were conducted to explore the impact of retinoblastoma  on the health status of survivors.  Content analysis was used for analysis and the International Classification of Functioning  Disabilities and Health (ICF) was used as a framework. | 156 Retinoblastoma survivors aged 8–35 years. | Of all survivors, 55% perceive RB-related restrictions in daily life activities (school, professional career, mobility, self-care, intimate relationships).  Young/adolescent survivors (6%) and adult survivors (15%)  frequently report anxiety about developing a second primary tumor (SPT). Compared with the general population, RB survivors did not differ in rates of employment or marital status. However, special  educational services were more frequently offered, and the level of completed education was lower. | 6/18  Low |
| 50 | KENNESARENMALM2009 | Sweden | Grounded theory | Guided by grounded theory methodology, the study explored the  main concerns of women with recurrent breast cancer, and how they were dealing with their situations. Data were collected from 40 in-depth interviews with 20 women diagnosed with recurrent breast cancer. | 20 women diagnosed with recurrent breast cancer. | The core category  illustrated the process of “making sense of living under the shadow of death,” and was based on the women’s experiences of adjusting to living with a persistent life-threatening illness. Confronting a recurrence of breast cancer was a  life-altering event. Moving through a difficult and challenging time, women eased their distress by letting go of losses and reassessing important values. | 12/18  Medium |
| 51 | KJORVEN HAUG2014 | Norway | Not reported | The aim of the study was to explore how  older people with incurable cancer experience the existential  meaning-making function in daily living from a life-span perspective. 21 participants (12 men and 9 women), aged 70–88, were interviewed in a  semi-structured framework. The selective optimization with compensation  (SOC) model from life-span developmental psychology was used deductively to explore the participants’  life-oriented adaptive strategies. | 21 older people with incurable cancer (12 men and 9 women), aged 70–88. | 1. Existential meaning making 2. Continuation of Goals and Focus on the Most Important goals 3. Plasticity and a Variety of Means and Resources Invested in Reaching the Most Important Goals 4. Reframing of Loss and Growth Leading to More Realistic Goal Adjustments | 10/18  Medium |
| 52 | MISSEL2011 | Denmark | Phenomenology | The study explored how patients diagnosed with incurable oesophageal cancer experience living with the illness.  The method takes a phenomenological-hermeneutic approach. The stories materialise from narrative  interviews, and the phenomena of the patients’ life world results in an analysis of these stories. | Five patients between the ages of 43 and 76 years with incurable cancer. | Themes :   1. Disease debut, denial and suspicion 2. Existential turning point, despair and hope 3. The body, affirmation of irrevocable illness 4. Acknowledging death and the significance of life phenomena 5. Social relations and feeling independent | 8/18  Medium |
| 53 | HAGGLUND  2015 | Sweden | Not reported | The objective of the study is to describe the lung cancer care process as experienced by patients, as well as to perform a  qualitative analysis of problems they encounter throughout the patient journey.  A user-centered design approach was used and data collected through two focus group meetings  with patients. The first stage of the process was to do an initial analysis and description of the current patient journey. The initial patient  journey model was based on literature and materials gathered  from clinicians at the Karolinska hospital who are involved in different stages of the lung cancer diagnosis and treatment  process. The model was then presented to the patients to validate that we had indeed captured the stages that are  important for the patients. Two focus group interviews were held to explore the problems and experiences of the patients and how these  problems change throughout the patient journey. Content analysis was used to identify categories and  themes related to the patient experience. | 9 patients with lung cancer were recruited via the lung cancer patient  organization Stödet (http://stödet.se) in Stockholm, Sweden. | The results indicate that not only is the patient journey fragmented and different for each patient going through it depending upon their specific type of lung cancer and treatment options, but their  experiences are also highly individual and dependent on their  personal needs and interpretations of the process.  5 distinct phases that the lung cancer patients go through: (1) pre-diagnosis care (primary and/or acute), (2) diagnostic examinations, (3) treatment, and finally (4) rehabilitation (when the patient is in remission), or (5)  palliative care.  Describing the process in terms of phases and events is important, but designers of eHealth services also need guidance when patients experience problems:  Unnecessary delays before  diagnosis, Poor communication  with health care, poor coordination, Understanding  of procedures, patient follow-up, Poor support for multimorbidity, Learning to manage one’s care, Lack of channels for giving  feedback, Understanding  of rights to choose. | 8/18  Medium |
| 54 | APPLETON  2015 | UK | Grounded theory | The aim was to explore how men cope with prostate cancer, its treatment and the strategies they employ to manage their diagnosis alongside daily life. | Twenty-seven men were interviewed at different stages in their disease pathway: nine men prior to radiotherapy, eight men at 6–8 months post radiotherapy and 10 men at 12–18 months post radiotherapy. | Regardless of the point at which they were interviewed four areas emerged as important to the men: the pathway to diagnosis; the diagnosis; the impact of prostate cancer and its treatment on daily life; and living with prostate cancer. | 12/18  Medium |
| 55 | CAYLESS2009 | UK | Grounded theory | Three serial interviews  were conducted at (1) diagnosis, (2) treatment and (3) follow-up. A longitudinal prospective qualitative study exploring the experiences of people in the first year following a diagnosis of prostate cancer was conducted. | The experiences of ten men diagnosed with prostate cancer. | The results are presented in three main sections focusing on how people manage their biographies and the disruption that cancer causes to health and hoped-for futures. Notions of biography and identity weave their way through men's accounts of prostate cancer. Physical side effects and reconstructed futures each form key parts of men's narratives.  Accepting and expecting a prostate cancer diagnosis.  Treatment: effects and disruptions, Projecting disrupted futures. | 9/18  Medium |
| 56 | ERVIK2010 | Norway | Phenomenology | The aim of this study was to illuminate men’s experiences living with localized or local  advanced prostate cancer when curative treatment such as surgery or radiation therapy is not an option at the time of diagnosis. The study was conducted via qualitative interviews, using a phenomenological hermeneutic approach. | Ten men treated with endocrine therapy or under active surveillance were interviewed. | Six core themes were identified. Getting ‘‘cancer’’ was the main  theme to which the other themes related; the other themes  included being hit in ‘‘the strength,’’ being on one’s own, not feeling worthy of treatment, a ‘‘silent’’ health care system, and  obtaining the best support. | 10/18  Medium |
| 57 | JONSSON  2010 | Sweden | Phenomenology | The aim of this qualitative study was to provide information if and how prostate cancer affects  men’s daily lives 2 years after the diagnosis. A second follow-up interview with men who were diagnosed with localized or advanced prostate cancer  approximately 18–24 months earlier. | Twenty-two men (aged 50–85 years) with prostate cancer participated. | Three equivalent fusions which influenced the men are: ‘Age is claiming its due’, ‘Living  with uncertainty’, and ‘Strengthen self-esteem’. The unifying fusion is identified as ‘Balancing a changed life situation.’ | 8/18  Medium |
| 58 | CARTER2004 |  | Grounded theory | The aim of the study was to explore what people living with  terminal illness considered were the areas of priority in their  lives. | Ten people living with terminal cancer were interviewed. | Living with dying: five inter-related themes (personal/intrinsic factors, external/extrinsic factors, future issues, perceptions  of normality and taking charge) encompassing the issues of importance to all participants. Each theme focused on ‘life and living’ in relation to life as it was or would be without illness. Practical issues of daily living and the opportunity to address philosophical issues around the meaning of life emerged as important areas.  The central theme, ‘taking charge’, concerned with people’s levels of life engagement, was integrally connected to all other themes. | 11/18  Medium |
| 59 | DAVIES2002 | UK |  | The aim of this  research was to develop a theory to explain the meaning and experience of living with advanced breast cancer. Grounded theory methodology was used to conduct  semi structured interviews. | Ten patients with advanced breast cancer i.e. in the palliative stage | Five key themes were identified:   1. Reconciling a different me 2. Time bomb 3. Media effect 4. Professional Waning 5. Rescuers   With Reconciling a different me being the overarching theme combining all others | 11/18  Medium |
| 60 | HARLEY2015 | UK |  | Semi Structured interviews  A priori themes from DOH were applied: clinical support; self-care and self-management; supporting independence; psychological  support; social and economic factors | Advanced cancer  Chronic Cancer: Patient Experience and self-management needs  56 patients> 12 months post diagnosis for advanced cancer | For patients to do well in this cancer phase requires good self-management of symptoms plus  taking an active role in accessing appropriate services  as needed.  Care planning at the point of transition to the chronic phase of cancer should focus on evaluating  patients’ needs, clarifying support pathways, increasing the profile and involvement of community services and  organisations, and supporting patients and families develop effective self-management skills. | 15/18 High |
| 61 | HOULDIN  2006 | USA |  | To describe the experiences of patients living with newly diagnosed stage III or IV colorectal cancer.  Semi-structured interviews were recorded on audiotape. Interviewers asked participants to describe their experiences  with the diagnosis and treatment of colorectal cancer. Content analysis  with inductive coding was used to code the transcribed interview data.  Categories were reviewed and organized into larger groupings, from which the core category was derived. | 14 patients newly diagnosed with advanced (stage III or stage IV) colorectal cancer. | Six domains: feeling life  is disrupted, experiencing physicians, feeling unprepared for everything, rethinking parenting, wondering “why me?,” and dealing with it. The core  category that explained study participants’ experiences with recently diagnosed colorectal cancer was “salvaging their normal lives.”  The dominant experience of the study participants focused on four aspects of their illness experience: (a) framing it in ways  that enabled them to recreate a semblance of normalcy or of their pre-illness state, (b) trying to tell children about the illness in stabilizing ways, (c) generating or maintaining a positive outlook no matter what, and (d) concretely managing the distress of the illness and its symptoms. | 11/18  Medium |
| 62 | LA COUR2009 | Denmark |  | Interpretative Approach based on Ricoeur’s theory of narrative emplotment    Of  Qualitative Interviews  And  Participant Observations | Advanced cancer  Breast Colon and Lung Cancer patients    Activity and meaning making in patients with Advanced Cancer  Purposeful sample of 7 | Key findings   - Saying goodbye in a good way - “Being healthy although ill” - Creating “my little Mecca” of life confirming experiences   Routines and continuity as a platform for agency | 12/18 Medium |
| 63 | LEVY2015 | UK | Phenomenology | This study explores men with advanced prostate cancers’ own  practices for promoting and maintaining emotional well-being using Interpretative Phenomenological Analysis. | Five men with advanced prostate cancer participated in face-to-face, semi-structured, in-depth interviews. | Living with an imminent and uncertain death. Temporality and the future self, Uncertainty and loss of control, Separation from life, Holding on to life, Containing and revealing emotions, Striving towards the future, Living in the now, Taking care of the family, Renegotiating purpose, two super-ordinate themes emerged – ‘living with an imminent and uncertain death’ and  ‘holding on to life.’ | 9/18  Medium |
| 64 | MAHER2011 | UK | Phenomenology | The aim was to explore the experience of living with relapsed Multiple Myeloma for eight patients accessing treatment within a haematology unit in a large London hospital. data were collected through open-ended, unstructured interviews. | 8 patients with multiple myeloma (incurable) | Findings suggest that living with relapsed myeloma in the context of a chronic illness causes an ever-shifting perspective between illness and wellness  consequently maintaining a state of uncertainty.  Overarching theme: Living with uncertainty  Intuitive knowing  Maintenance of normalcy  Adjustment to illness  Hope  Effects of treatment  Trusting healthcare professionals  Fighting spirit  Receiving the bad news | 9/18  Medium |
| 65 | NISSIM2012 | Canada | Grounded theory | A longitudinal qualitative research study was undertaken to provide an understanding of a prolonged experience of advanced cancer, as seen through the eyes of dying individuals. The authors conducted a total of  54 interviews with these patients to learn of their experience of advanced cancer. | 27 patients with either advanced lung or gastrointestinal cancer  who had an expected survival of up to 2 years. Advanced cancer | Core category: striving to grow in  the land of the living/dying, symbolizing their sense of finding themselves in a borderland between life and death where their efforts focused on 3 common goals:  controlling dying, valuing life in the present, and creating a living legacy. | 10/18  Medium |
| 66 | REED2015 | UK |  | This study set out to systematically explore  the illness trajectory of metastatic breast cancer  using models from chronic illness as a  framework. A longitudinal  mixed-methods approach was adopted. Using a  primarily qualitative approach, a number of data sources were combined to systematically chart the  illness trajectory of breast cancer from the point of  diagnosis of progressive disease until death. Women were interviewed three times over one calendar year using a qualitative narrative approach. Data from women’s narrative  interviews and documentation (including medical notes, oncologist annotations of appointments,  nursing notes, correspondence between healthcare professionals, investigation results) were triangulated  to map in detail the illness trajectory of each woman’s metastatic breast cancer. | Ten women aged between 40 and 78 years, with metastatic breast cancer. | Five trajectory  phases were identified; the pre-trajectory phase, trajectory  onset, living with progressive disease, downward  phase and dying phase. | 7/18  Medium |
| 67 | REEVE2009 | UK |  | A qualitative study  with a purposive sample of 19 primary palliative care patients. In-depth interviews were analysed using the iterative thematic analysis | Advanced cancer | Living with cancer can be an exhausting process.  Maintaining continuity of everyday life was the norm, and dependent on a dynamic  process of balancing threats and supports to people’s emotional well-being. Interactions  with health care professionals were therapeutic when they provided emotional, or narrative, support. Threats arose when the patient’s perception of the professional’s account of their illness experience was at odds with the person’s own sense of their core self and what was important to them. Our findings highlight the need for a framework in which clinicians may legitimately utilize different illness models to deliver a personalized, patient-centred assessment of need and care. | 7/18  Medium |
| 68 | REEVE2010 | UK |  | Holistic Form Analysis | Terminal cancer  19 patients with terminal cancer [within last 6 months of life], identified through GP surgeries  Semi structured interviews | Two distinct narrative forms   1. Continuity 2. Fracture | 17/18 High |
| 69 | SINDING2002 | Canada | Phenomenology | IPA framework interviews with women with breast cancer. In this study, the researchers consider performance in its hermeneutic  sense, as a way of generating meaning. The drama Handle With Care? Living With Metastatic Breast Cancer was created by a research team, a theatre troupe, and women  with breast cancer. explored what it means to have metastatic disease and what it means to provide oncological care to women with this diagnosis. | Women with metastatic breast cancer (n=4) | Experience of the disease disrupting daily life. Her illness becomes something she takes hold of, something she grasps  differently from before. The meaning of metastatic disease as anguish, as profound and unpredictable disruption, is not erased. the possibility of recurrence as insistent. Women with breast cancer are both exposed and enclosed, both laid open to danger and trapped by their diagnoses. The meanings of metastatic breast cancer that  emerged depicted advanced disease as disturbing, intrusive, and unmanageable. | 3/18  Low |
| 70 | WINTERLING  2004 | Sweden |  | There are few studies on patients’ perceptions of their situation after being  recently diagnosed with an advanced gastrointestinal cancer. | Fourteen patients with advanced gastrointestinal cancer were interviewed. | All informants perceived substantial changes in life. This  included negative physical, mental, and practical changes as well as positive changes. Mental changes included 3 categories: despair, why, and uncertainty.  The informants described several ways of handling these changes in life. The most frequently reported by patients were that “one shouldn’t complain” and by  spouses to “hope,” and by all informants to “make the best of it.” Other ways of handling the situation were reconciliation, avoidance, preparation for death,  seeking support, and isolation. | 8/18  Medium |
| 71 | DE GUZMAN  2013 | Philippines | Grounded theory | The study sought to conceptualize the process of cancer survivorship among Filipinos. The researchers used a two-part instrument to  surface the process of acceptance, fortitude, and recovery among adult cancer survivors. The first part, is the subject’s *robotfoto*,  a Dutch term defined as “a cartographic sketch of  the subject” (Kelchtermans & Ballet, 2002) to establish  the baseline characteristics of the cancer survivors under study. It consists of circles and blank spaces to obtain needed information. The second  part is a focus group interview intended to elicit the respondents’ own experiences with regard to their path to recovery. | Twenty-seven Filipino cancer survivors were purposively selected13 respondents previously diagnosed with breast cancer, another for the 11 who had colon cancer, and a third for the remaining 3 participants. | The findings describe a model:  the *trifling* (living before), *transfusing* (accepting the reality), *transforming* (being strong), and *transcending*  (living beyond) phases of cancer survivorship. Ten sub-stages were also identified: *tainting, desolating, disrupting,* and *embracing* for the transfusing phase; *tormenting*, *distressing*, *awakening*, and *transfiguring* for the transforming phase, and *trembling* and *enlivening* for the transcending phase. | 12/18  Medium |
| 72 | FOLEY2006 | USA | Framework approach | The aim of the study was to elucidate meanings ascribed to the cancer experience by long-term survivors. Semi-structured interviews were conducted with 58 cancer survivors (>15 years post-diagnosis).  Respondents described how cancer affected their quality of life (QOL) generally and in 17 domains. Systematic  content analyses were conducted to extract themes relating to meanings assigned to the cancer experience. Themes were analysed by cancer type, gender, and age and confirmed using quantitative assessments of self-rated health and  QOL. | Across Cancer types | Four themes were identified: Personal Growth, That’s Life. Relinquishing Control, and Resentment.  Women more frequently acknowledged Personal Growth, and men more often indicated minimal impact on their  lives (That’s Life). Older survivors were disproportionately classified as That’s Life and younger survivors as Personal Growth. No differences were observed by cancer type or ethnicity. | 10/18  Medium |
| 73 | HUBBARD  2011 | UK |  | Mixed Interpretative Methods of written accounts of cancer experience | Cancer  Cancer as biographical disruption  40 patients | Key Findings:   - Cancer is a biographically disruptive with ongoing physical and psychosocial impacts - Cancer survivors should be given opportunities to articulate the   impact of cancer, thus giving legitimate space to talk about  cancer’s ongoing resonance on life so that problems and  difficulties are not dismissed or trivialised. | 12/18 Medium |

**Online data Supplement 4: The ARC Framework themes and sub-themes**


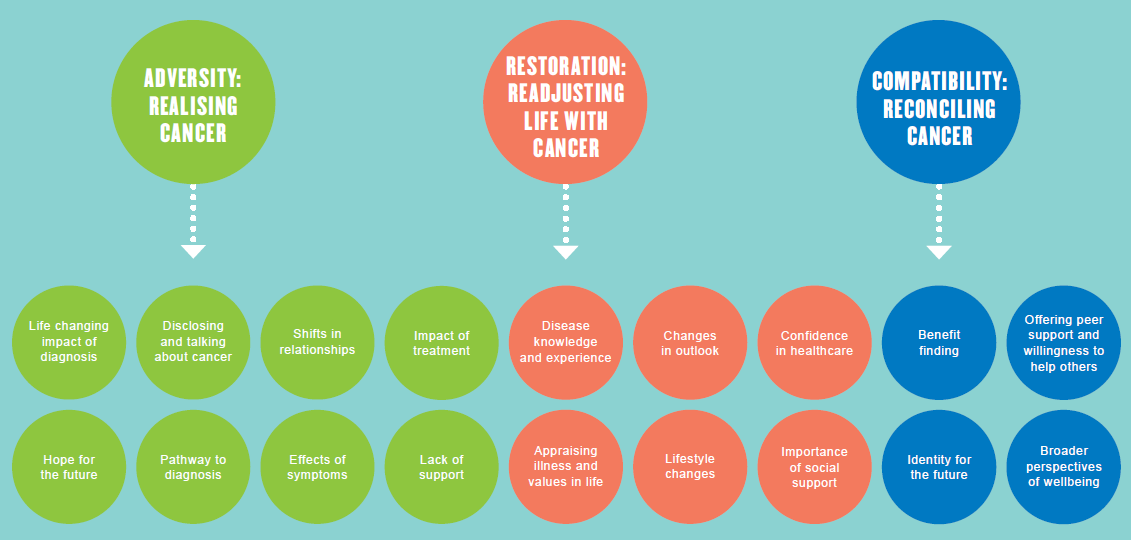


**Online data Supplement 5: Full coding framework**

**Theme 1: Adversity: Realising cancer**

- 1. Pathway to diagnosis
     1. *Delayed diagnosis*
     2. *Misdiagnosis*
     3. *Receiving the diagnosis (how it is communicated)*
  2. Life changing impact of diagnosis
     1. *Realisation of mortality*
     2. *Challenge to identity and sense of self*
     3. *Heightened awareness of the body*
     4. *Concerns of recurrence*
     5. *Hope for a future life*
  3. Disclosing and talking about Cancer
     1. *Reveal or conceal*
     2. *Stigma and embarrassment*
  4. Effects of symptoms (Consequences of a problematic body)

*1.4.1 Physical changes ‘the body broken and torn’*

*1.4.2 Emotional chaos*

*1.4.3 Loss of functional independence*

*1.4.4 Social well-being*

*1.4.5 Financial distress*

- 1. Impact of treatment
     1. *Physical changes*

*1.5.1.1 Body image and changes in appearance*

- - 1. *Emotional relationship with the body*

*1.5.2.1 Coping with effects caused by treatment*

- - 1. *Practical concerns: a whole different lifestyle*
       1. *Disrupted everyday roles*
    2. *Social interactions*
    3. *Managing health care needs*
       1. *Appointments, scans and check-ups*

1.6 Lack of support

*1.6.1* *Lack of social support*

*1.6.2 Health care disconnect*

*1.6.2.1 Accessing health care support*

*1.6.2.2 Unmet information needs*

*1.6.2.3 Loss of support in post-treatment phase*

*1.6.3 Lack of available support for others*

1.7 Shifts in relationships (inc work relationships)

*1.7.1 Burdening others*

*1.7.2 Social isolation*

*1.7.3 Impact of Providing Support for others*

*1.7.4 Changes in intimacy*

**Theme 2: Restoration: Readjusting life with cancer**

2.1 Disease knowledge and experience

- - 1. *Access to information*
       1. *Accuracy of information*
  1. Appraising illness and values in life
     1. *Finding an explanation for illness*
     2. *Striving for normality*
     3. *Challenging societal attitudes to cancer*
  2. Changes in outlook
     1. *Distress and resilience*
     2. *Focus beyond illness*
     3. *Hope for an ordinary life*
  3. Lifestyle changes

*2.4.1 Goal setting*

*2.4.2 Choosing meaningful activities*

*2.4.3 Maintaining socially valued roles*

- 1. Importance of social support
     1. *Relationships with family and friends*
     2. *Peer support and support groups*
     3. *Support from healthcare providers*

*2.5.4 Non-discriminatory work places*

*2.5.6 Community support*

*2.5.6.1 Religion & spirituality*

- 1. Confidence in health care
     1. *Value of specialist staff*
     2. *Participation in treatment planning*
        1. *Partnership in decision-making*
     3. *Need for psychological support*

| **Theme 3: Compatibility: Reconciling cancer**  3.1 Benefit finding  *3.1.1 Improved self-esteem*  *3.1.2 Better relations and sense of connection*   - - 1. *Finding opportunities for personal growth*   3.2 Offering peer support and willingness to help others  3.3 Broader perspectives of wellbeing   - - 1. *Greater appreciation of life*     2. *Shift in priorities*     3. *Organising life in a different way*   1. Identity for the future   *3.4.1 Adapting to physical and functional changes*  *3.4.2 Feeling psychologically stronger*  *3.4.3 Regaining roles* |
| --- |

**Online data Supplement 6: Vote counting of ARC sub-themes**

| Study # | ADVERSITY: REALISING CANCER | | | | | | | RESTORATION: READJUSTING LIFE WITH CANCER | | | | | | COMPATIBILITY: RECONCILING CANCER | | | |
| --- | --- | --- | --- | --- | --- | --- | --- | --- | --- | --- | --- | --- | --- | --- | --- | --- | --- |
|  | Pathway to diagnosis | Life changing impact of diagnosis | Disclosing and talking about cancer | Effects of symptoms – physical, emotional, practical, social, financial | Impact of treatment – physical, emotional, practical, social, financial | Lack of support- social, health care | Shifts in relationships | Disease knowledge and experience | Appraising illness and values in life | Changes in outlook | Lifestyle changes | Importance of social Support –family, friends, peers, healthcare | Confidence in health care | Benefit finding | Offering peer support and willingness to help others | Broader perspectives of well-being | Identity for the future |
| 1 |  |  |  | X | X | X |  |  |  |  |  | X |  | X | X |  |  |
| 2 |  |  |  | X | X |  |  |  |  |  | X |  |  |  |  |  |  |
| 3 |  | X |  | X | X | X |  | X | X |  | X | X | X |  | X |  |  |
| 4 |  | X |  | X | X | X |  |  |  |  |  | X | X |  |  |  |  |
| 5 |  | X |  | X |  | X |  | X | X | X |  | X | X | X | X | X | X |
| 6 |  | X |  |  | X |  |  | X |  |  |  |  |  | X |  |  |  |
| 7 |  |  |  |  |  |  |  |  | X | X | X | X | X | X |  |  |  |
| 8 |  | X |  | X | X | X |  |  |  |  |  |  |  |  |  |  |  |
| 9 |  | X |  | X | X | X |  |  | X |  |  |  |  | X |  | X | X |
| 10 |  | X |  |  | X | X |  |  |  |  |  |  |  |  |  |  | X |
| 11 |  | X |  | X |  | X |  |  | X |  |  | X |  |  |  |  | X |
| 12 |  | X |  | X | X | X | X |  |  | X | X | X |  |  |  |  |  |
| 13 |  | X | X | X | X | X |  |  |  |  |  |  |  | X |  |  | X |
| 14 |  | X |  |  | X | X |  |  | X |  |  |  |  |  |  |  |  |
| 15 |  | X |  | X | X |  |  | X |  |  | X | X | X |  |  |  |  |
| 16 |  | X |  |  | X |  |  |  | X | X | X |  |  |  | X |  |  |
| 17 |  |  |  |  |  |  |  |  | X | X | X | X |  | X |  |  |  |
| 18 |  |  |  |  | X | X |  |  | X | X | X | X |  |  |  |  |  |
| 19 |  | X | X | X | X | X |  |  | X | X | X | X |  | X | X |  |  |
| 20 |  | X |  | X | X |  |  |  |  |  | X | X |  |  |  | X |  |
| 21 |  | X |  |  | X |  |  |  | X |  | X |  |  |  |  |  |  |
| 22 |  | X | X | X |  | X |  |  |  |  |  |  |  |  |  |  |  |
| 23 |  | X |  |  |  | X |  |  |  |  |  |  |  |  |  |  |  |
| 24 | X | X | X |  | X | X |  | X |  |  |  | X |  |  |  |  |  |
| 25 |  | X | X |  |  | X |  |  |  |  |  | X | X |  |  |  |  |
| 26 | X | X |  | X | X | X |  |  | X | X | X | X |  | X |  |  |  |
| 27 |  | X |  | X | X | X | X |  | X | X | X |  |  |  |  | X |  |
| 28 |  | X | X | X | X |  |  | X | X | X |  |  |  | X | X | X | X |
| 29 |  | X | X | X | X | X |  | X |  |  |  | X |  |  |  |  |  |
| 30 |  | X |  | X |  |  |  | X | X | X | X |  | X |  |  |  |  |
| 31 |  | X | X | X | X | X | X |  | X | X | X | X |  | X | X | X |  |
| 32 |  | X |  | X | X |  |  |  |  |  |  | X |  | X | X |  |  |
| 33 | X | X |  | X |  | X |  | X | X |  |  |  |  |  |  |  |  |
| 34 | X |  | X | X | X | X |  | X |  |  |  |  |  |  |  |  |  |
| 35 | X | X | X | X | X | X | X |  |  |  |  | X |  |  |  |  |  |
| 36 |  |  |  |  |  |  |  |  | X | X | X | X | X | X |  | X | X |
| 37 |  | X |  |  |  | X |  |  |  |  |  |  | X |  |  |  |  |
| 38 |  | X |  |  |  | X |  |  |  | X |  |  |  |  |  |  |  |
| 39 |  | X | X | X | X | X |  |  |  |  |  |  |  |  |  |  |  |
| 40 |  |  |  | X | X | X | X |  |  |  |  |  |  |  |  |  |  |
| 41 |  | X | X | X | X | X | X |  |  |  |  |  |  |  |  |  |  |
| 42 |  |  |  | X | X |  |  |  |  |  | X |  | X |  |  |  |  |
| 43 |  | X |  |  | X | X | X |  |  |  |  |  |  |  |  |  |  |
| 44 | X | X |  | X |  |  | X |  |  |  |  | X | X |  |  |  |  |
| 45 |  | X |  |  | X | X |  |  | X | X | X |  |  |  | X |  |  |
| 46 | X | X |  |  | X |  |  |  |  |  | X |  |  |  |  |  |  |
| 47 |  | X |  | X | X |  |  | X | X |  |  | X |  |  |  |  |  |
| 48 |  |  |  | X | X |  |  |  |  |  |  | X |  | X |  |  |  |
| 49 |  | X |  | X |  |  |  |  |  |  |  |  |  |  |  |  |  |
| 50 |  | X |  |  |  |  | X |  | X |  |  |  |  |  |  |  |  |
| 51 |  | X |  |  |  |  |  |  | X |  | X |  |  |  |  |  |  |
| 52 |  | X |  | X | X |  |  |  |  |  |  | X |  |  |  |  |  |
| 53 | X | X |  |  | X | X |  |  |  |  |  |  |  |  |  |  |  |
| 54 | X | X |  | X | X |  |  |  |  |  | X | X |  |  |  |  |  |
| 55 |  | X |  |  | X |  |  | X |  |  | X |  |  |  |  |  |  |
| 56 |  | X |  |  |  | X | X |  |  |  |  | X |  |  |  |  |  |
| 57 |  |  |  |  |  |  |  | X |  |  | X |  |  |  | X | X |  |
| 58 |  |  |  | X | X | X | X |  |  |  |  |  |  |  |  |  |  |
| 59 |  | X |  |  |  | X | X |  |  |  |  | X |  |  |  | X |  |
| 60 | X |  |  | X | X | X | X |  |  | X |  |  |  |  |  |  |  |
| 61 |  |  |  | X | X | X |  |  |  | X | X | X |  |  |  |  |  |
| 62 |  | X | X |  |  |  |  |  |  |  | X |  |  |  |  |  |  |
| 63 |  | X | X | X |  |  |  | X | X |  | X |  |  |  |  |  |  |
| 64 |  | X |  |  | X | X | X |  |  |  |  |  |  |  |  |  |  |
| 65 |  | X |  |  | X |  |  |  |  |  |  |  |  |  |  |  |  |
| 66 | X | X |  |  |  | X |  |  |  |  |  |  |  |  |  |  |  |
| 67 | X | X |  |  |  | X |  |  |  |  |  | X | X |  |  |  |  |
| 68 |  |  |  | X | X | X | X |  | X |  |  | X |  |  |  |  |  |
| 69 |  | X |  |  |  |  |  |  | X |  |  |  |  |  |  |  |  |
| 70 |  | X |  | X | X | X |  |  | X | X |  |  |  |  |  |  |  |
| 71 |  | X |  |  |  |  |  |  | X | X | X |  |  |  | X | X |  |
| 72 |  | X |  |  | X |  | X |  |  | X | X |  |  | X |  | X |  |
| 73 |  | X |  | X | X |  | X |  |  | X |  |  |  |  |  |  |  |
|  | 12 | 58 | 14 | 41 | 48 | 42 | 17 | 14 | 27 | 21 | 28 | 30 | 12 | 15 | 11 | 11 | 7 |
